# 3-D ocean particle tracking modeling reveals extensive vertical movement and downstream interdependence of closed areas in the northwest Atlantic

**DOI:** 10.1038/s41598-020-76617-x

**Published:** 2020-12-08

**Authors:** S. Wang, E. L. Kenchington, Z. Wang, I. Yashayaev, A. J. Davies

**Affiliations:** 1 Department of Fisheries and Oceans Canada, Bedford Institute of Oceanography, 1 Challenger Drive, Dartmouth, NS B2Y 4A2 Canada; 2 Department of Biological Sciences, Center for Biotechnology and Life Sciences, University of Rhode Island, 120 Flagg Road, Kingston, RI 02881 USA

**Keywords:** Marine biology, Physical oceanography, Ecological networks

## Abstract

Novel 3-D passive particle tracking experiments were performed in the northwest Atlantic to elucidate connectivity among areas closed to protect vulnerable marine ecosystems. We examined (1) the degree of vertical movement of particles released at different depths and locations; (2) the location of potential source populations for the deep-sea taxa protected by the closures; and (3) the degree of functional connectivity. A long-term oceanographic dataset (EN4) was queried to characterize the temperature and salinity regimes in each of the closed areas as a basis for interpreting recently published climate change projections. Using the Parcels Lagrangian particle tracking framework and the BNAM hydrodynamic model, we found enhanced connectivity over previously developed 2-D models and unexpected, current-driven, strong (to a maximum of about 1340 m) downward displacement at depth (450, 1000 and 2250 m), with weaker upward displacement except for the release depth of 2250 m which showed upward movement of 955 m with a drift duration of 3 months. The current velocities create down-stream interdependence among closed areas and allow redundancy to develop in some of the areas of the network, with some of the larger areas also showing retention. Source populations for sponges in the upstream closure are likely in adjacent waters of the Canadian continental shelf. Collectively this information can be used to inform management decisions related to the size and placement of these closed areas, and vertical velocity surfaces have potential for use in species distribution modeling of benthic species and habitats.

## Introduction

The large-scale distributions of deep-sea invertebrate species are strongly associated with hydrographic features, largely the water mass characteristics of temperature and salinity^1,2^. This relationship has underpinned the adoption of species distribution models to predict the presence, abundance or biomass of these species through both space and time^3^. However, at local scales, distribution patterns are more strongly influenced by topographically interacting hydrodynamic processes such as currents, downwelling and other mechanisms^4^. Understanding how the distribution of species are influenced at varying scales is a priority for conservation and management of vulnerable marine species. Multiple tools and approaches are available, but most recently Lagrangian particle tracking (LPT) has emerged as an important tool for assessing structural connectivity in the deep sea^5–8^. In such models, virtual particles are advected by the flow fields generated by numerical models^9^, and virtual behavior can be added to the particles so that they can act as active drifters, i.e., larvae. When combined with hydrodynamic data, this tool has great potential to enhance our understanding of species distributions and to predict functional connectivity.

**Figure 1:**
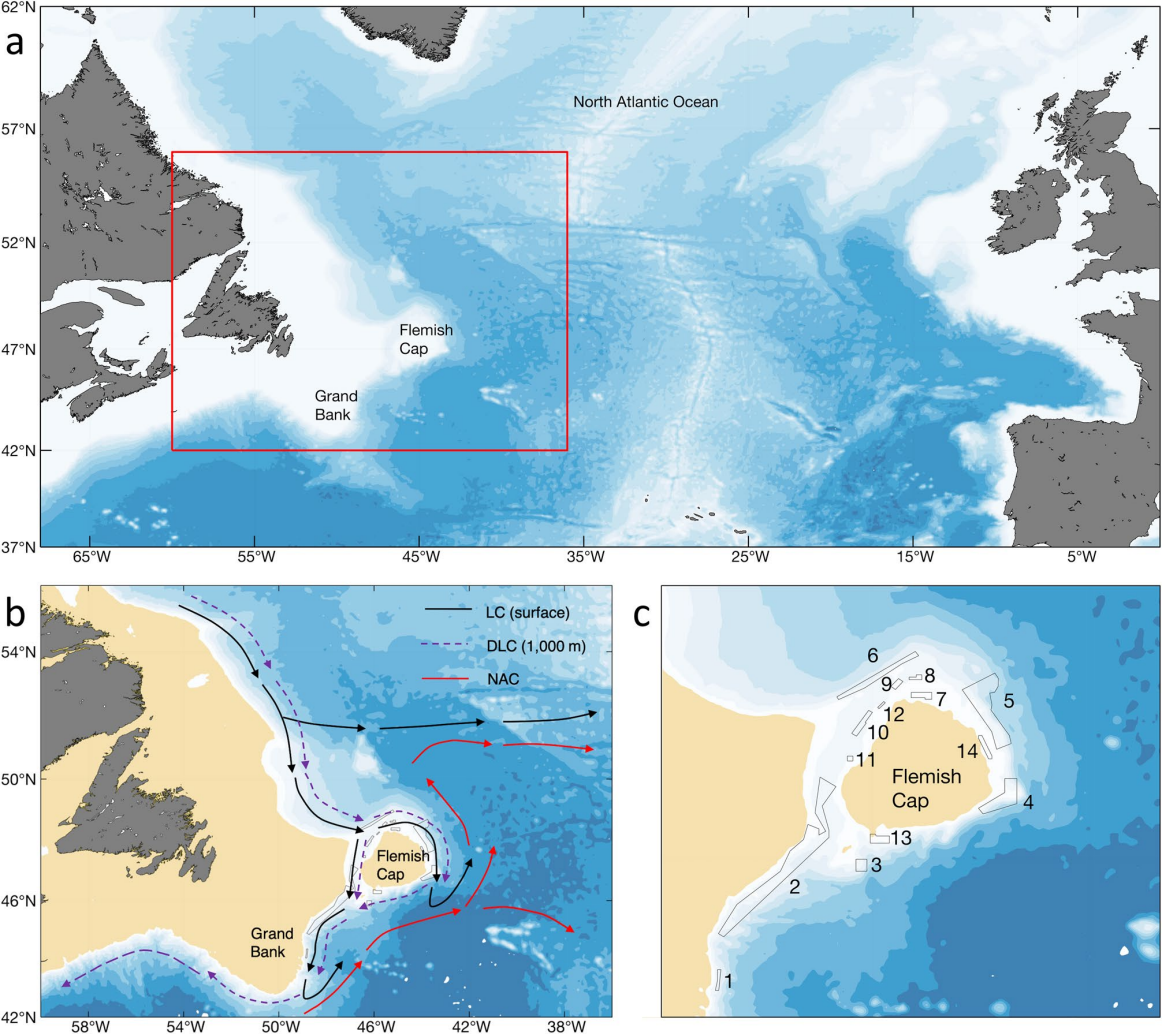
Maps Detailing the Study Area. (**a**) The position of Flemish Cap and Grand Bank within the North Atlantic; (**b**) inset from (**a**) (red outline) showing a schematic diagram for the pathways of the major currents in the region: the Labrador Current (LC) at the surface, the Deep Labrador Current (DLC) at 1000 m and the North Atlantic Current (NAC); (**c**) locations of the 14 NAFO closed areas on Flemish Cap and the Tail of Grand Bank and their associated area codes. The maps were generated using bathymetry and coastline data produced and made publically available by the NOAA National Centers for Environmental Information (NCEI). The ETOPO1 Ice Surface (https://doi.org/10.7289/V5C8276M) arc-minute global relief model of the Earth’s surface (https://www.ngdc.noaa.gov/mgg/global/) was used to generate bathymetry and the Global Self-consistent, Hierarchical, High-resolution Geography Database (GSHHG; https://www.ngdc.noaa.gov/mgg/shorelines/gshhs.html) was used to produce co-ordinates for a high-resolution coastline and both plotted using Matlab version 9.5 software (https://www.mathworks.com) with the M_Map mapping package (version 1.4 m, created by R. Pawlowicz, https://www.eoas.ubc.ca/~rich/map.html).

The northwest Atlantic contains a diverse array of benthic habitats^10–14^, and although 285 epibenthic invertebrate taxa have been identified from this region to date^13^, conservation efforts have largely focussed on vulnerable marine ecosystems (VMEs) characterized by fragile, long-lived species that are vulnerable to impacts by bottom-contact fishing gears^15^. The regional fisheries management organization operating in this area beyond national jurisdiction, the Northwest Atlantic Fisheries Organization (NAFO), has closed 14 areas on the Flemish Cap and the Tail of Grand Bank (Fig. 1) to protect deep-sea corals, sea pens and sponges^16^. Closed Areas 1–6 were closed primarily to protect deep-sea sponge grounds, Areas 7–12 and 14 were primarily closed to protect sea pens, and Areas 2, 4, 5 and 13 to protect large gorgonian corals^7^. These closed areas occur in deep water ranging from 483 m (Area 2) to 2754 m depth (Area 4). While place-based management measures can offer protection from direct impacts of bottom-contact fishing gears, the long-term viability of the protected populations will depend on identifying and protecting sources of recruitment and connectivity pathways^17^.

In the Flemish Cap region where most of these closures are found (centered around 47° N, 45° W with a 200 km radius at the 500 m isobath), two major currents, the Labrador Current (LC) and the North Atlantic Current (NAC) carry water masses with different origins^18,19^. The LC, including its deep branch, carries Denmark Strait Overflow Water (DSOW), Labrador Sea Water (LSW), and Iceland-Scotland Overflow Water (ISOW) from the north into this region, while the NAC transports warm and saline Gulf Stream water from the south. The bathymetry of the slopes creates topographic obstacles to water flows that strongly affects the currents (Fig. 1). The LC bifurcates to the northwest of Flemish Cap, one branch flowing through the Flemish Pass and the other circulating around Flemish Cap in a clockwise direction (Fig. 1). In the surface waters the LC interacts with the NAC to the east and south of Flemish Cap. Given this complex hydrographic regime, it is not surprising to find that regional species distribution models for deep-sea sponges, large gorgonian corals, and sea pens all identify hydrographic variables as important drivers of occurrence^7,10,11,13^.

Kenchington et al.^7^ used a 2-D LPT model to evaluate structural and functional connectivity among the 14 areas that were closed to protect vulnerable marine ecosystems by NAFO. They found that the regional pattern of currents led to downstream interdependence between some of the closed areas. A system of weakly-connected closed areas to protect sea pen VMEs on Flemish Cap was identified and species distribution models were used to evaluate potential source areas. That work had various limitations^20^, most notably particle tracking was limited to relatively shallow depth zones using vertical averages of the velocity fields for each depth interval modeled: 0–5 m (surface) and 95–105 m (100 m), and particle tracking was only run with horizontal movement (2-D). Here, we build on that work by applying 3-D LPT models to assess connectivity among closed areas put in place by NAFO. Our experiments are used to simulate larval transport and show how temporal and spatial ocean model changes affect dispersal. We performed three different simulation experiments aimed at determining different aspects of biophysical connectivity among the 14 closed areas. We specifically examine: (1) the degree of vertical movement of particles released at different depths and locations directed by the position of the closed areas; (2) the location of potential source populations for the deep-sea sponges, large gorgonian corals, and sea pens protected by the closed areas; and (3) the degree of functional connectivity between the closed areas taking into consideration the spawning times of the VME indicator species present. Lastly, a long-term oceanographic dataset (EN4) was queried to characterize the temperature and salinity regimes in each of the closed areas as a basis for interpreting climate change projections^21^.

## Results

### Vertical movements in 3-D models

**Figure 2:**
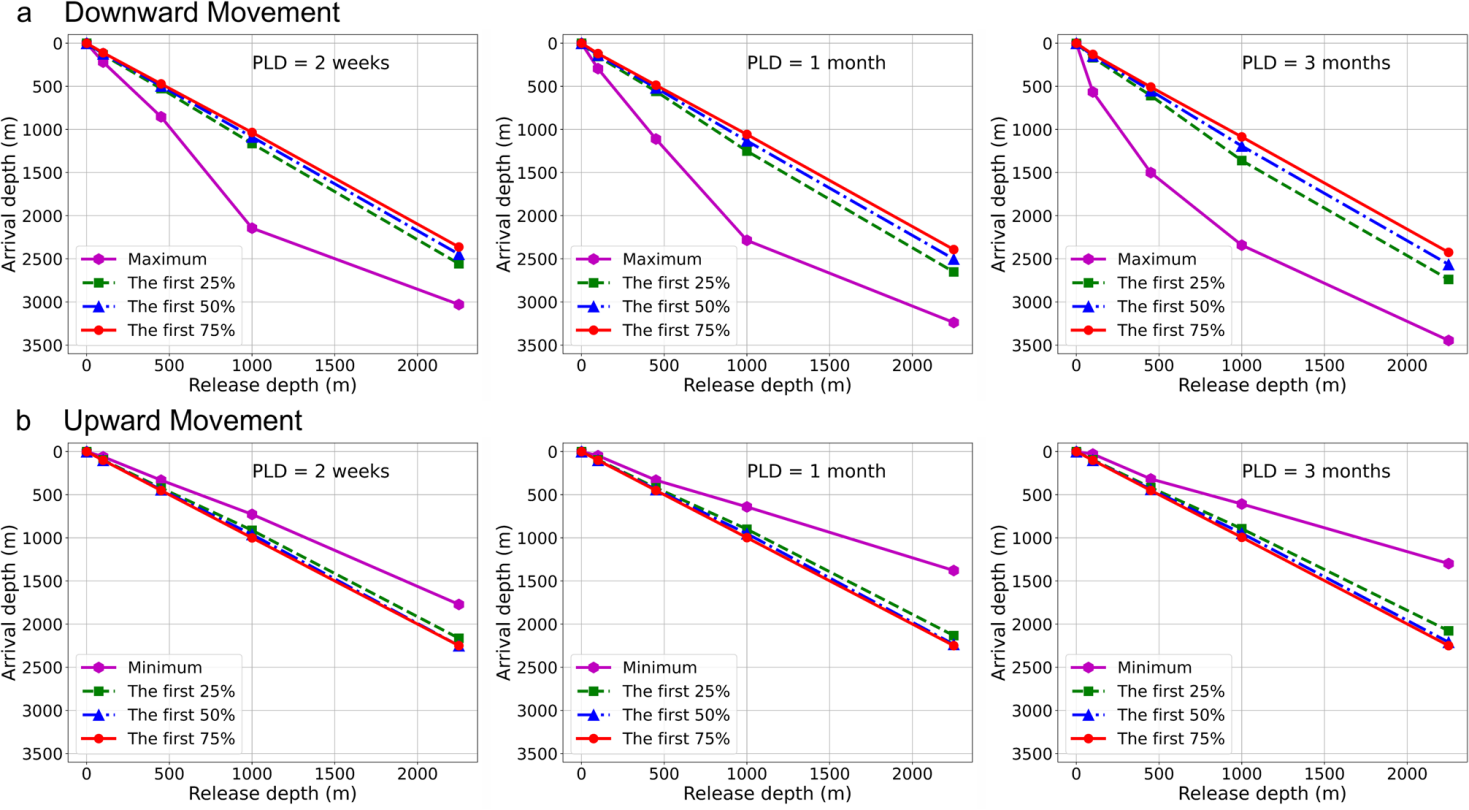
Vertical Particle Movements. (**a**) downward and (**b**) upward minimum depths that forward-tracked particles reached under each of 3 pelagic larval drift durations (PLD) using 3-D models and the average current for 5 particle release depths (surface, 100 m, 450 m, 1000 m and 2250 m). The depths for which the arrival of the first 25% (green lines and symbols), 50% (blue lines and symbols) and 75% (red lines and symbols) of particles are shown for each scenario.

Particles released at the surface generally stayed within close proximity to the surface under all time durations due to strong surface currents leading to rapid horizontal dispersion of the particles (Table 1, Fig. 2). Particles released at 100 m stayed near that depth unless left for 3 months at which time the maximum depth reached showed strong downward movement of over 400 m (Fig. 2a, Table 1). However, the maximum depth reached by 50% of the particles was only 146.8 m (Table 1), indicating that the particles showed greater dispersion at this depth and duration. The vertical movement of particles showed stronger downward maximum vertical movements for passive particles released at 450 m, 1000 m and 2250 m depths for all 3 drift durations (2 weeks, 1 month and 3 months) (Table 1, Fig. 2a). Particles released at 1000 m and 2250 m showed the strongest downward movement (Table 1) with maximum displacement of over 1000 m evident even under a one-month drift duration (Table 1). However, the maximum depth reached by 50% of the particles was always less than 300 m at these release depths and durations (Table 1).

**Table 1:** Vertical particle displacements.

Particle release depth	Drift duration	Minimum depth reached (m)	Minimum depth reached by the first 50% of particles (m)	Maximum depth reached (m)	Maximum depth reached by the first 50% of particles (m)
Surface	2 weeks			0.78	0.55
	1 month			1.40	0.59
	3 months			2.90	0.69
100 m	2 weeks	59.7	100.0	218.9	121.9
	1 month	47.4	100.0	294.3	136.9
	3 months	28.1	99.7	565.3	146.8
450 m	2 weeks	331.0	441.8	853.0	494.7
	1 month	331.0	439.5	1110.6	517.8
	3 months	316.9	437.3	1500.7	549.9
1000 m	2 weeks	727.1	960.7	2144.1	1087.1
	1 month	640.9	951.3	2285.6	1132.1
	3 months	607.5	947.0	2341.8	1191.5
2250 m	2 weeks	1771.9	2250.0	3029.8	2446.4
	1 month	1378.6	2235.1	3238.5	2502.4
	3 months	1298.2	2209.8	3446.4	2565.1

Depths for particles released under different model depth and drift duration scenarios to examine vertical movement. All scenarios used forward-tracked 3-D models and the average current for five particle release depths (surface, 100 m, 450 m, 1000 m and 2250 m). The minimum and maximum depths for the termination of the first 50% of particles are provided.

Upward movement was generally not as extensive as the downward (Table 1, Fig. 2b), with maximum upward displacements of less than ~ 75 m at a release depth of 100 m, less than ~ 135 m at release depth of 450 m, less than ~ 395 m at release depth of 1000 m, and less than ~ 955 m at a release depth of 2250 m (Table 1); all were achieved under longer drift durations. For 50% of the particles, the minimum depth reached was similar to that of the release depth.

**Figure 3:**
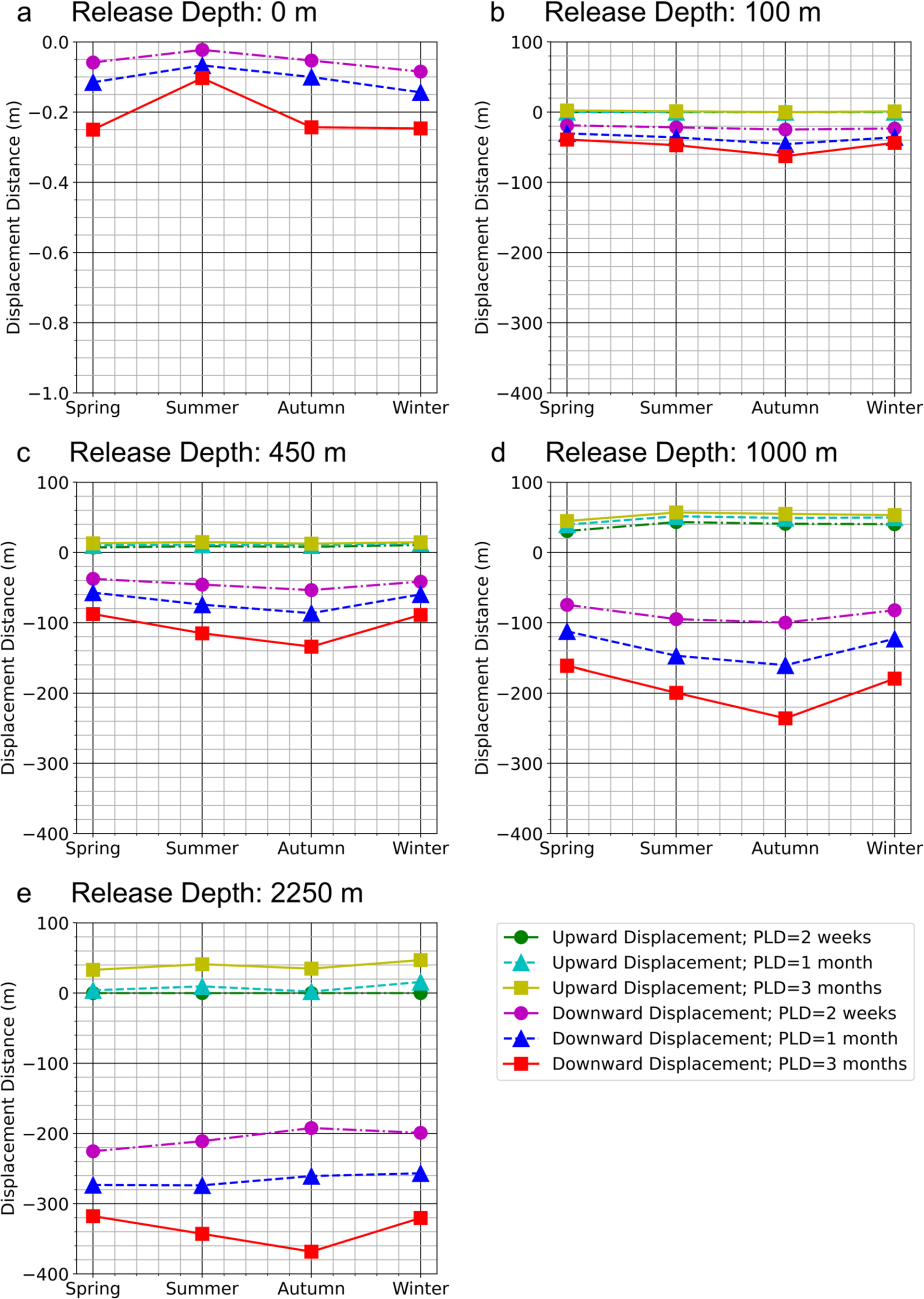
Seasonal Vertical Particle Displacements. Upward and downward travelling distances in each of four northern hemisphere seasons with different particle release depths ((**a**–**e**) Surface, 100 m, 450 m, 1000 m and 2250 m) and pelagic larval drift durations (PLD; 2 weeks, 1 month and 3 months) in terms of the depths at which 50% of the forward-tracked particles released where able to reach. Positive and negative values denote upward and downward movements, respectively.

Both the upward and downward displacement by the 25%, 50%, and 75% of particles was much less pronounced than the minimum and maximum depths reached (Fig. 2, Table 1), indicating that the majority of particles typically stayed in the vicinity of their release depth (Table 1). Particles released at 100 m and 450 m stayed at that depth or slightly deeper within 1 month duration, but if left for 3 months they also showed downward displacement of around 70 m and 150 m for 25% particles at each depth respectively (Fig. 2). Particles released at 1000 m and 2250 m showed the strongest downward movement with a displacement of around 360 m and 500 m with 3-month drift duration for 25% particles at each depth respectively (Fig. 2). Upward movement was not as great as the downward, with upward displacements of 25% particles around 100 m (release depth 1000 m) and 180 m (release depth 2250 m) (Figs. 2a, 3). A seasonal evolution of flow pattern is known to exist around Flemish Cap^22,23^. Downward movements by the first 50% of sorted particles remained prevalent for all seasons and release depths evaluated, with little vertical movement at the surface and 100 m water depth (Fig. 3a,b). Increasingly prominent downward movements were observed in deeper water. At 450 m, 1000 m and 2250 m release depths, seasonality appeared to impact vertical travelling distances that were greater in the summer and autumn than at other seasons (Fig. 3c–e).

These results show that even though the majority of particles stayed at their release depths, there is potential for significant vertical displacement of passive particles in this region and that seasonally applied connectivity experiments with 3-D modelling are needed to give an improved interpretation of particle trajectories over models that only take into account horizontal movements^7^.

### Identification of potential source populations

**Figure 4:**
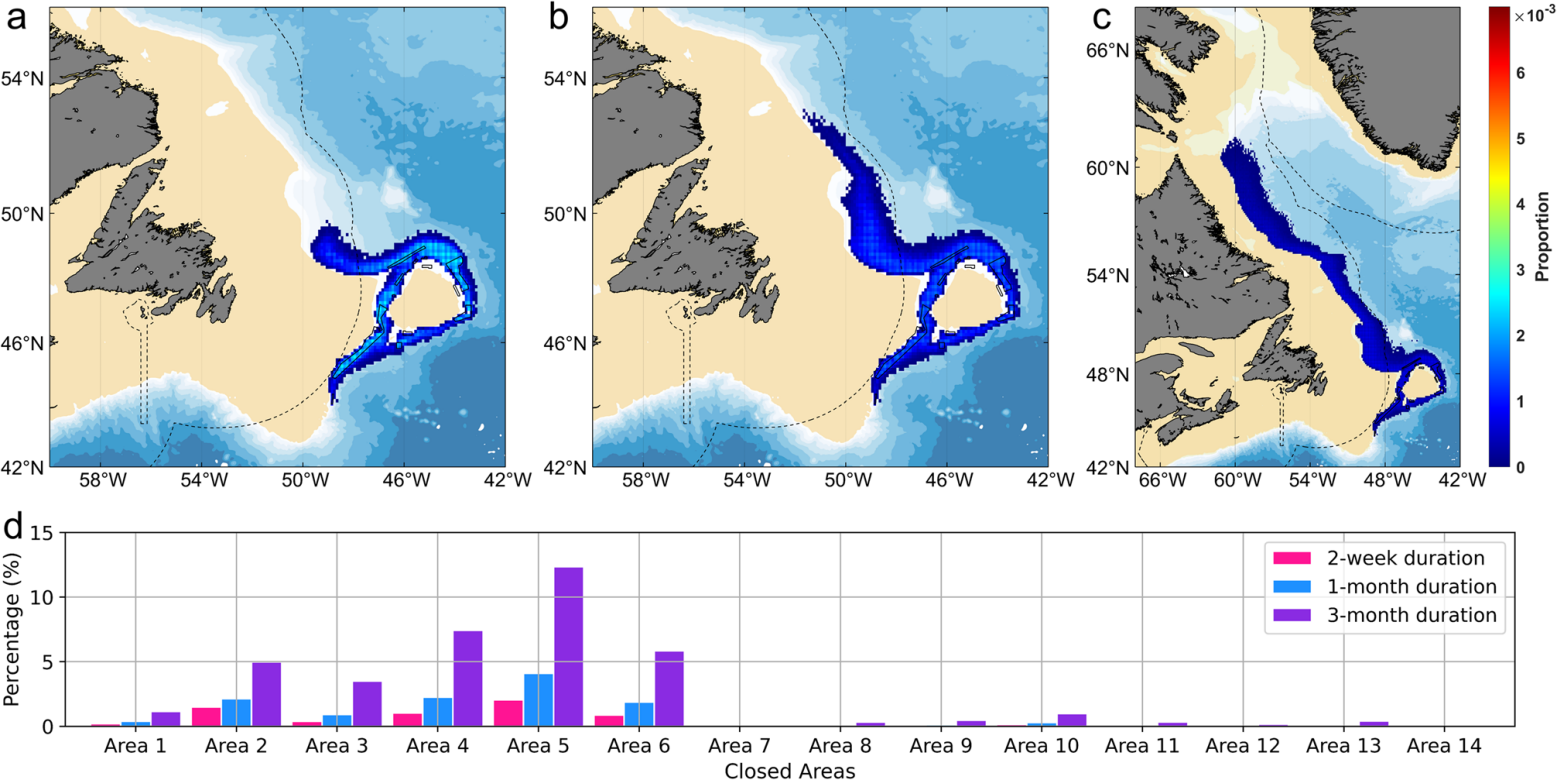
Potential source populations. Spatial distribution of the proportion of released particles ending in and passing over each 0.1° × 0.1° grid cell with particles released at 1000 m depth and forward-tracked with drift durations of: (**a**) 2-week, (**b**) 1-month and (**c**) 3-month drift durations; (**d**) the proportion of released particles (1000 m) entering each closed area (see Fig. 1c) under three drift duration scenarios. Dashed lines indicate boundaries of national jurisdictions downloaded from the Maritime Boundaries Geodatabase, version 11, available online at https://www.marineregions.org/, https://doi.org/10.14284/382. The maps were generated using bathymetry and coastline data produced and made publically available by the NOAA National Centers for Environmental Information (NCEI). The ETOPO1 Ice Surface (https://doi.org/10.7289/V5C8276M) arc-minute global relief model of the Earth’s surface (https://www.ngdc.noaa.gov/mgg/global/) was used to generate bathymetry and the Global Self-consistent, Hierarchical, High-resolution Geography Database (GSHHG; https://www.ngdc.noaa.gov/mgg/shorelines/gshhs.html) was used to produce co-ordinates for a high-resolution coastline and both plotted using Matlab version 9.5 software (https://www.mathworks.com) with the M_Map mapping package (version 1.4 m, created by R. Pawlowicz, https://www.eoas.ubc.ca/~rich/map.html).

Results from particles released at 1000 m (which approximated the mean depth across all closed areas) over the entire spatial domain with particles passing over and ending inside one of the 14 closed areas (Fig. 4a–c) showed that deep-sea species around Flemish Cap likely have source populations to the north, inside Canadian waters, that were transported by the Deep Labrador Current (DLC) along the continental slope, consistent with our models of vertical displacement at this release depth (Figs. 2, 3). The proportions of particles reaching the closed areas (Fig. 4d) were higher for the areas with larger size (Areas 2, 4, 5 and 6) and highest for Area 5. Almost no particles entered Area 7 and 14 within a 1-month drift duration due to their maximum depths (718 and 688 m respectively) relative to the 1000 m release depth in this simulation. Increasing drift duration increased the proportion of particles arriving in all closed areas, as would be expected. The results of the models where particles were released from the closed areas and back-tracked, show a similar spatial distribution (Supplementary Fig. S1) to that produced from the forward models seeded over the full spatial domain at the same 1000 m depth (Fig. 4), although the proportion of particles reaching another closed area is much higher, as expected from the position of the release sites. Area 2 shows a higher proportion of particles in the back-tracked model for the 2 week duration, reflecting retention in this large closed area (Supplementary Table S5), which reduced in proportion at the 1 month and 3 month durations as particles left the closed area (Supplementary Fig. S1).

### Functional connectivity of sponges, sea pens and large Gorgonian corals

#### Large-sized sponges

**Figure 5:**
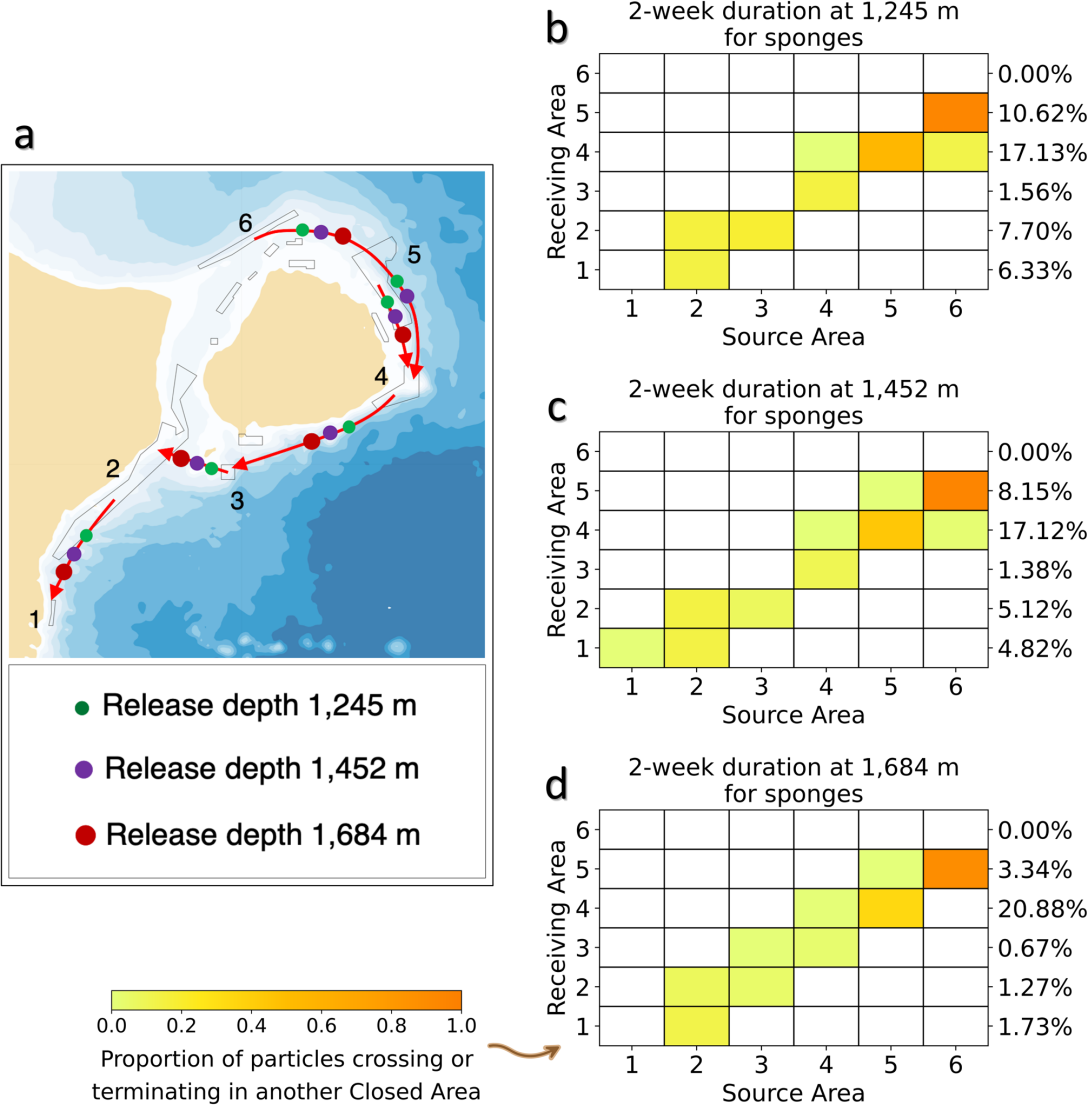
Functional connectivity among areas closed to protect sponges. (**a**) Forward-tracking connectivity pathways for particles released at 3 depths in each of the 6 areas closed to protect large-size sponges (Areas 1–6) showing chain-linking and minimal redundancy in Area 4. Drift durations were 2 weeks. The closed circles over the lines indicate particles can reach another area when released from this depth. The map was generated using bathymetry and coastline data produced and made publically available by the NOAA National Centers for Environmental Information (NCEI). The ETOPO1 Ice Surface (https://doi.org/10.7289/V5C8276M) arc-minute global relief model of the Earth’s surface (https://www.ngdc.noaa.gov/mgg/global/) was used to generate bathymetry and the Global Self-consistent, Hierarchical, High-resolution Geography Database (GSHHG; https://www.ngdc.noaa.gov/mgg/shorelines/gshhs.html) was used to produce co-ordinates for a high-resolution coastline and both plotted using Matlab version 9.5 software (https://www.mathworks.com) with the M_Map mapping package (version 1.4 m, created by R. Pawlowicz, https://www.eoas.ubc.ca/~rich/map.html); (**b**–**d**) the proportion of modeled particles released from each of the 6 areas closed to protect large-sized sponges (Source Areas; Areas 1, 2, 3, 4, 5, 6) and passing over or terminating in another area closed to protect large-size sponges (Receiving Areas). For each Receiving Area the percentage of the total number of particles released (from all source areas) are provided. Those values include particles that crossed, terminated or were retained in the Receiving Area. Drift durations were 2 weeks using average currents from the ocean model. Release depths were 1245 m, 1422 m and 1684 m respectively.

The connectivity amongst areas closed to protect sponges (Areas 1–6) was assessed after a pelagic larval duration (PLD) of 2 weeks, which approximates the larval duration of deep-sea sponges^24^. Dominated by the Deep Labrador Current, a clockwise connection around Flemish Cap was observed in both forward- and back-tracking models with downstream interdependence and minimal redundancy (Fig. 5a, Supplementary Fig. S2). Six connections between areas closed to protect sponges were observed with models using average oceanographic values, at the minimum and middle depths of the mean depth ranges for the combined areas, while only 5 connections were observed at the maximum depth where the connection between Area 6 and Area 4 was not found in the forward-tracking model but was picked up in the backward-tracking model (Supplementary Table S1). A seventh connection between Area 5 and Area 3 was made in summer only at 1245 m. Retention within a closed area increased with depth and was observed in Areas 1, 2, 3, 4, 5, and 6 at different depths/seasons, with greater retention being identified in the backward-tracking models (Supplementary Table S1). Area 6 had downstream links with Area 5 and Area 4 for all three concerned depths. No particles released from Area 6 reached Area 4 at the release depth of 1684 m in the forward-tracking models (Fig. 5) although the back-tracking model picked up a much higher proportion of particles in Area 6 (Supplementary Fig. S2, Supplementary Table S1). There was potential for Area 5 to connect with Area 4, thereby conferring some redundancy for Area 4. It is very likely that Areas 4, 3 and 2 make connections with Area 3, 2 and 1, respectively (Fig. 5, Supplementary Fig. S2, Supplementary Table S1). These connections had relatively short arrival times of 10 days or less (Supplementary Fig. S5). Particles released from Area 5 at 1245 m depth reached another closed area (likely Area 4) peaking at 6 days, while complimentary particles backtracked from Area 4 showed a peak transit time of 4 days to Area 5 (inferred; Supplementary Fig. S5). The downstream chain-linking of the closed areas is evident in Fig. 5a and Supplementary Fig. S2. A large proportion of the particles released in Area 6 connect to Area 5 at all depths simulated (Fig. 5b–d; Supplementary Fig. S2). However, the redundancy in Area 4 results in it receiving the greatest percentage of the total particles released and forward-tracked at each depth scenario, ranging from 17.13% at 1245 m to 20.88% at 1684 m. In comparison, the connection between Area 2 and Area 1 area was relatively weak in the forward-tracking model with only a small proportion of particles making the connection. However, the higher number of particles seeded in the back-tracking model allowed for identification of Area 2 as an important source of particles for Area 1 (Supplementary Fig. S2). It should be noted that Area 1 has a small spatial area, which is possibly one reason for the reported weak connectivity in the forward modeling; this closed area does not appear as a potential Source Area in the back-tracked models as there is no downstream closed area to act as a destination for particles released from Area 1 (Supplementary Fig. S2). Area 6, being the furthest upstream area in the network (Fig. 5a) is similarly unconnected as a Receiving Area in the forward-tracking models due to its position, and has only minimal retention in the deepest parts of the closure identified only in back-tracking models (Supplementary Table S1), making this area highly dependent on source populations from outside of the network (Fig. 4). The connectivity matrices were significantly correlated between Summer, Autumn and the Average backward-tracking models for all three depths with Pearson’s r-values of 0.997–0.999, indicating statistically similar results across seasons and averaged models at each release depth.

#### Sea pens

**Figure 6:**
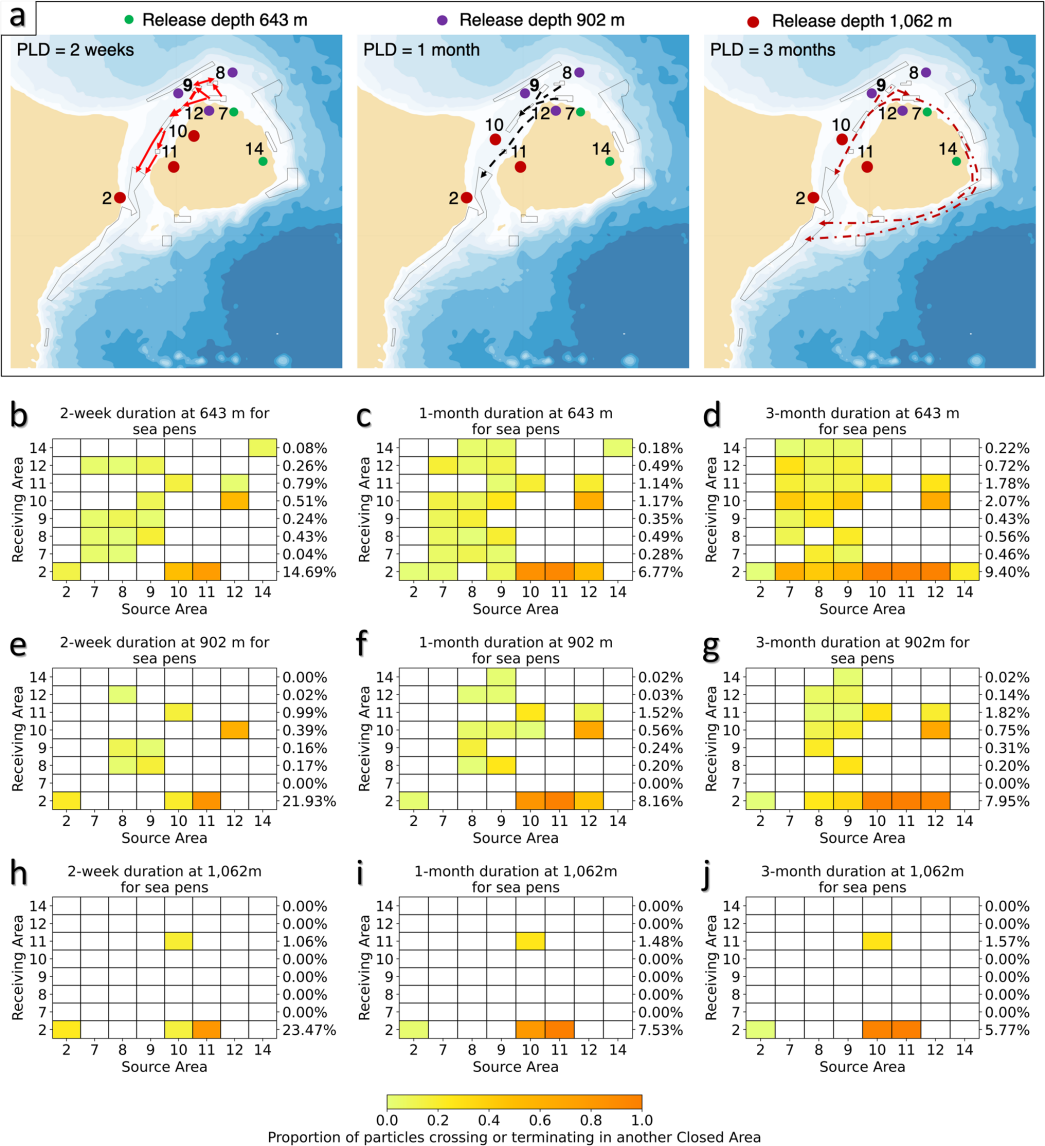
Functional connectivity among areas closed to protect sea pens. (**a**) Forward-tracking connectivity pathways for particles released at 3 depths in each of the 8 areas closed to protect sea pens (Areas 2, 7, 8, 9, 10, 11, 12, 14). Drift durations were 2 weeks, 1 month, and 3 months. The closed circles near the number code of some of the closed areas denotes that particles from this area are only released at this depth due to the depth of the closure, and the line indicates where particles can reach another closed area when released from this depth. The maps were generated using bathymetry and coastline data produced and made publically available by the NOAA National Centers for Environmental Information (NCEI). The ETOPO1 Ice Surface (https://doi.org/10.7289/V5C8276M) arc-minute global relief model of the Earth’s surface (https://www.ngdc.noaa.gov/mgg/global/) was used to generate bathymetry and the Global Self-consistent, Hierarchical, High-resolution Geography Database (GSHHG; https://www.ngdc.noaa.gov/mgg/shorelines/gshhs.html) was used to produce co-ordinates for a high-resolution coastline and both plotted using Matlab version 9.5 software (https://www.mathworks.com) with the M_Map mapping package (version 1.4 m, created by R. Pawlowicz, https://www.eoas.ubc.ca/~rich/map.html); (**b**–**j**) The proportion of modeled particles released from each of the 8 areas closed to protect sea pens (Source Areas; Areas 2, 7, 8, 9, 10, 11, 12, 14) and passing over or terminating in another area closed to protect sea pens (Receiving Areas). For each Receiving Area the percentage of the total number of particles released (from all Source Areas) are provided. Those values include particles that crossed, terminated or were retained in the Receiving Area. Particle release depths were 643 m (**b**–**d**), 902 m (**e**–**g**) and 1062 m (**h**–**j**) with drift durations of 2 weeks (**b**,**e**,**h**), 1 month (**c**,**f**,**i**), and 3 months (**d**,**g**,**j**).

The connectivity amongst areas closed to protect sea pens (Areas 2, 7–12, and 14) was assessed after 2-week, 1-month and 3-month durations, as there is no consensus on PLD for this group^7^. These eight areas are located in the shallower waters of the Flemish Cap. Strong connections appear to exist among Areas 10, 11 and 12 resulting from their spatially proximate locations (Fig. 6). The back-tracking model showed a very high proportion of the particles originating in Area 10 connecting to Area 11, under all depth/duration scenarios (Supplementary Fig. S3), while the forward-tracking model showed similarly high proportions of particles connecting between Area 12 and Area 10, at 643 m and 902 m, with the latter being the Receiving Area (Fig. 6). A highly interconnected network operative was observed between Areas 7, 8 and 9 and Areas 10, 11 and 12 in both the back-tracked (Supplementary Fig. S3) and forward-tracked models (Fig. 6). As PLD increases to 1 month, connectivity appears to establish between more distant areas such as the connection from Areas 7, 8 and 9 with Area 2 (Fig. 6, Supplementary Fig. S3). After a 3-month PLD, long-term transportation may bring about the links from all the closed areas on Flemish Cap to Area 2 (Fig. 6, Supplementary Fig. S3). The currents are complex in this region with some particles displacing in a counterclockwise direction and clockwise in other locations. The minimum arrival time of particles making these connections was generally less than 1 month, while it may take more than 2 or 3 months for particles from Area 7 to reach Area 14 at 643 m. The transit time distributions for these areas present obvious fluctuations, which arise from the relatively large number of closed areas in close proximity to one another (Supplementary Fig. S5; only scenarios for 643 m/3 month PLD shown). Particles are very likely to arrive at these areas within 20 days drift time. The number of connections decreased sharply with depth; the depth ranges of these areas are small and release depths were close to the maximum depths of the areas, limiting the range of potential movement in the vertical. Those connections that were made generally contained a high proportion of released particles (Fig. 6, Supplementary Fig. S3). This is particularly evident for the connections to Area 2 from areas on the top of Flemish Cap and contribute to a high degree of redundancy for the sea pen populations of Area 2 (Supplementary Table S2), which were strongest from the shallower release depths and 3 month drift duration (Fig. 6). Area 2 received between 5.77 and 23.47% of the total number of particles released (Fig. 6). Retention occurred at all potential spawning seasons (spring, summer and winter) and on average in five of the eight areas with 2 week drift duration and decreased with particle release depth (Supplementary Table S2). Area 2, the largest of the areas, showed consistent retention of particles across drift durations, and release depths and to some extent across seasons (Supplementary Table S2). As for the sponges, connectivity matrices were significantly correlated between Spring, Summer, Winter and the Average backward-tracking models for all three depths with Pearson’s r-values ranging from 0.877 (643 m depth: Spring vs. Summer) to 0.999, indicating statistically similar results across seasons and averaged models at each release depth and drift time duration.

#### Large Gorgonian corals

**Figure 7:**
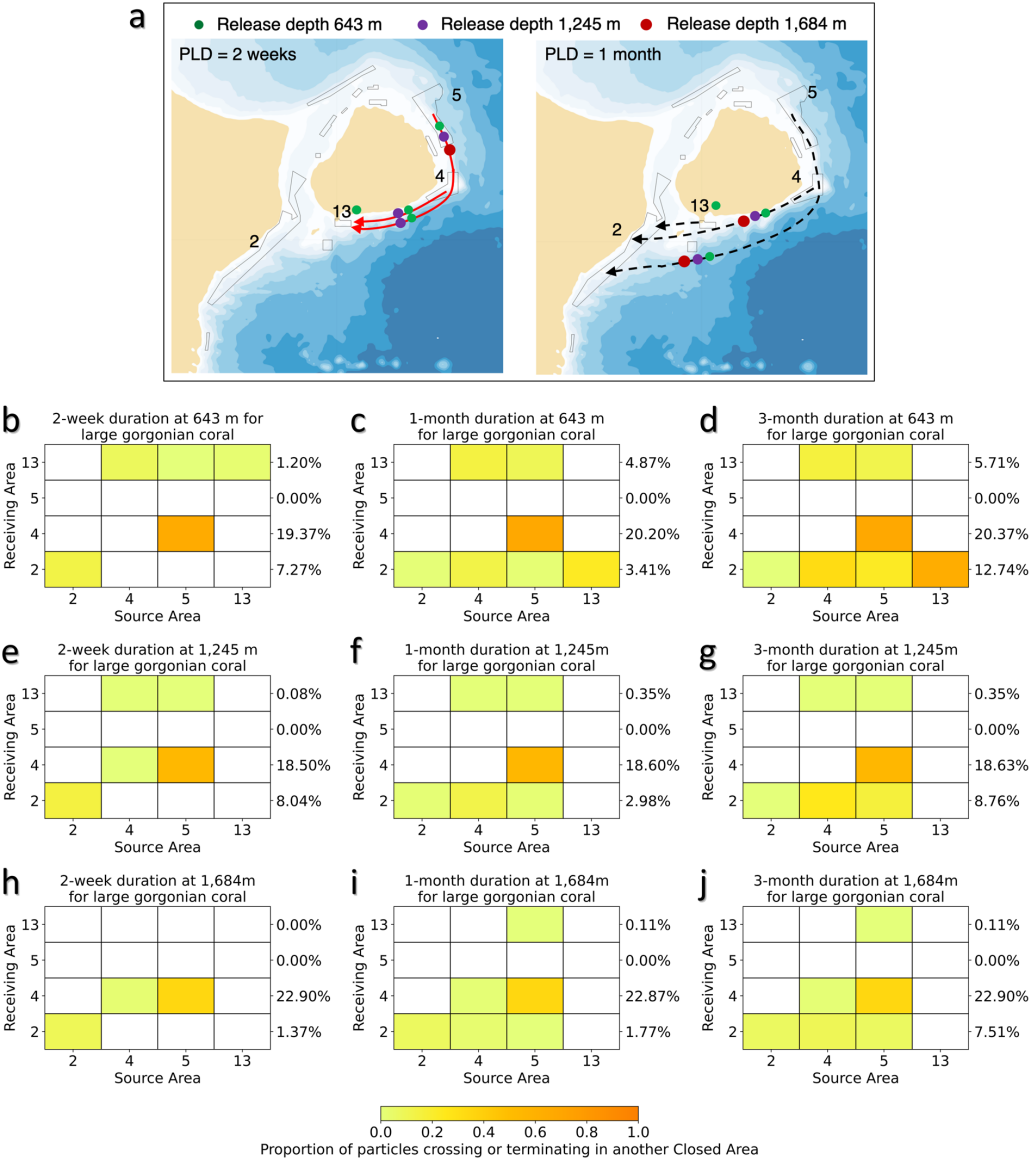
Functional connectivity among areas closed to protect large Gorgonian corals. (**a**) Forward-tracking connectivity pathways for particles released at 3 depths in each of the 4 areas closed to protect large gorgonian corals (Areas 2, 4, 5, 13). Drift durations shown are 2 weeks and 1 month. The connectivity map with 3-month drift duration is not shown because it is the same as that of 1-month drift duration. The closed circles near the number code of some of the closed areas denotes that particles from this area are only released at this depth due to the limitation of maximum depth, and the closed circles over the lines indicates particles can reach another closed area when released from this depth. The maps were generated using bathymetry and coastline data produced and made publically available by the NOAA National Centers for Environmental Information (NCEI). The ETOPO1 Ice Surface (https://doi.org/10.7289/V5C8276M) arc-minute global relief model of the Earth’s surface (https://www.ngdc.noaa.gov/mgg/global/) was used to generate bathymetry and the Global Self-consistent, Hierarchical, High-resolution Geography Database (GSHHG; https://www.ngdc.noaa.gov/mgg/shorelines/gshhs.html) was used to produce co-ordinates for a high-resolution coastline and both plotted using Matlab version 9.5 software (https://www.mathworks.com) with the M_Map mapping package (version 1.4 m, created by R. Pawlowicz, https://www.eoas.ubc.ca/~rich/map.html); (**b**–**j**) The proportion of modeled particles released from each of the 4 areas closed to protect large gorgonian corals (Source Areas; Areas 2, 4, 5, 13) and passing over or terminating in another area closed to protect large gorgonian corals (Receiving Areas). For each Receiving Area the percentage of the total number of particles released (from all Source Areas) are provided. Those values include particles that crossed, terminated or were retained in the Receiving Area. Particle release depths were 643 m (**b**–**d**), 1245 m (**e**–**g**) and 1684 m (**h**–**j**) with drift durations of 2 weeks (**b**,**e**,**h**), 1 month (**c**,**f**,**i**), and 3 months (**d**,**g**,**j**).

The connectivity amongst areas closed to protect large gorgonian corals (Areas 2, 4, 5 and 13) was assessed after 2-week, 1-month and 3-month PLD, as there is no consensus on larval duration for this group^7^. These areas evidenced a downstream clockwise connectivity in space at both 2-week and 1-month drift durations in the forward-tracking models (Fig. 7a,b, Supplementary Table S3) with the back-tracking models reinforcing downstream connectivity between Area 13 and Area 2 at 1245 m and 1684 m depths (Supplementary Fig. S4; Supplementary Table S3). There was some redundancy in Area 13 which receives particles from Areas 4 and 5 in both forward- and back-tracking models with the shorter drift duration and shallower particle release depths (Fig. 7, Supplementary Fig. S4, Supplementary Table S3). As the downstream closure, Area 2 demonstrated the greatest redundancy, receiving particles from all of the other areas (reaching a maximum of 12.74% of the total particles released in the forward-tracking model) (Fig. 7). This area also shows a degree of retention (Supplementary Table S3). However, the strongest connections were made between Area 5 and Area 4 with Area 4 receiving up to 22.90% of released particles from Area 5 in the forward-tracking models (including both retentions and particles passing over) and similarly high proportions under different drift duration and depth combinations (Fig. 7). Area 4 also demonstrated some retention of particles at the deeper release depth (Supplementary Table S3). The connection time between Area 5 and Area 4 peaks at approximately 5 days in both forward- and back-tracking modes at 643 m release depth (Supplementary Fig. S5), which may be too short for larval settlement.

### Water mass characteristics of the closed areas

The bottom temperature and salinity data from EN4 profiles in each closed area were averaged from all 12 months in each year, and shaded areas in the plot show the range of the data in each area within that year (Supplementary Fig. S6). The cruise data are averaged in each closed area for the year the survey was carried out and represent an independent data source. Not surprisingly, except for Area 12, the EN4 temperatures in all other six closed areas were ~ 1 °C lower than the cruise data. The coarse resolutions in horizontal and vertical spaces are likely the cause for this discrepancy. EN4 salinities were generally saltier than those reported from the cruise surveys. Though some discrepancy does exist, the EN4 data provide an opportunity to investigate the potential variability of temperature and salinity in each closed area. All fourteen areas had relatively stable salinity or temperature for the 1975–2017 period (Supplementary Fig. S6). The average bottom temperatures in these areas were all below 4 °C, with average salinity above 34.8 on the practical salinity scale (Supplementary Fig. S6), the reference value to calculate freshwater content/transport in many studies^25^. Areas 1–6, 10 and 14 were on average colder (below 3 °C) than Areas 7–9, 11–13, while salinity differed less between areas. We conclude that the epibenthic invertebrate species in these closed areas reside in a stable, cold and salty environment. Particles released from each of the closed areas and back-tracked for a period of 3 months also experienced stable temperature and salinity conditions over their pathways (not shown). Such particles released from Area 5 showed the greatest temperature variation in the standard deviation of the temperature along the pathway (< 0.31 °C), while Area 2 showed the greatest salinity variation (standard deviation < 0.054 PSU); all mean values of temperature and salinity were constant over the duration (not shown).

## Discussion

**Figure 8:**
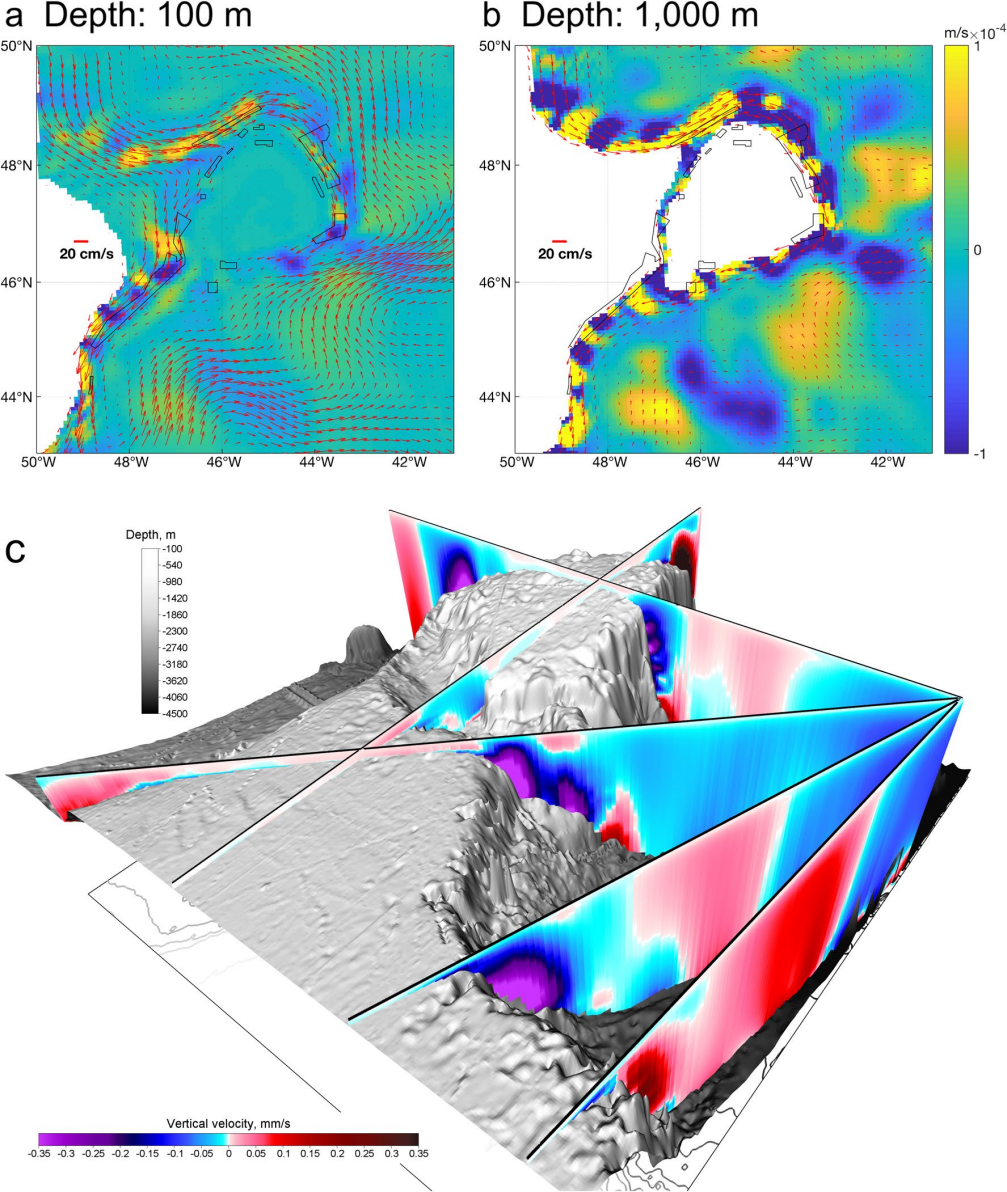
Distributions of horizontal velocity vectors (cm/s) and vertical velocity contours (m/s × 10^–4^). (**a**) 100 m and (**b**) 1000 m depth in the Flemish Cap area of the northwest Atlantic. Positive (yellow) and negative (blue) values denote upward and downward velocity respectively. (**c**) Vertical velocity slices showing areas of upward (reds) and downward (purples) velocity. The areas closed by NAFO to protect deep-sea sponges, corals and sea pens are outlined in black in (**a**,**b**). All data were derived from the BNAM oceanographic model. The maps in (**a**,**b**) were generated using bathymetry and coastline data produced and made publically available by the NOAA National Centers for Environmental Information (NCEI). The ETOPO1 Ice Surface (https://doi.org/10.7289/V5C8276M) arc-minute global relief model of the Earth’s surface (https://www.ngdc.noaa.gov/mgg/global/) was used to generate bathymetry and the Global Self-consistent, Hierarchical, High-resolution Geography Database (GSHHG; https://www.ngdc.noaa.gov/mgg/shorelines/gshhs.html) was used to produce co-ordinates for a high-resolution coastline and both plotted using Matlab version 9.5 software (https://www.mathworks.com ) with the M_Map mapping package (version 1.4 m, created by R. Pawlowicz, https://www.eoas.ubc.ca/~rich/map.html). The 3-D map in 8c was generated using the ETOPO1 Ice Surface bathymetry and plotted with Voxler software (https://www.goldensoftware.com/products/voxler).

Connectivity models are emerging as a useful tool for studies in the deep ocean (e.g.,^7,26^). However, their use, and by extension depiction of reality, relies upon the use of appropriate hydrodynamic models, a good understanding of the oceanographic conditions within the model domain, adequate testing of model parameters, and some knowledge of larval release and behavior. In this study, we extended earlier work^7,20^ by integrating a more complex larval dispersal model with a regional-scale hydrodynamic model capable of simulating 3-D particle movement. Ultimately, we observed enhanced connectivity among the NAFO closed areas and documented the potential for strong vertical displacement (Fig. 8) at release depths greater than 450 m (Figs. 2, 3) which encompassed the minimum depth of the closed areas to protect vulnerable species, that ranged from 483 to 2754 m^7^.

### Comparison with previous studies in the region

LPT models have previously been produced for the Flemish Cap region using two combinations of tracking and ocean models^7,20^. Model outputs from Parcels and BNAM^20^ and those generated with WebDrogue and the Quoddy ocean model^7^ were examined using like-for-like comparisons in 2-D applications^20^. Parcels/BNAM showed higher connectivity among the closed areas and a broader range of potential source areas than WebDrogue/Quoddy^20^. Such differences arise, in part, from the parameters used in the simulation, for example, greater number of simulated particles seeded in the Parcels/BNAM scenarios, their foundation in annual-mean currents and the incorporation of random walk. However, even when these factors were eliminated, differences still remained, suggesting that the underlying hydrodynamic models drive the differences in outputs^20^. Differences between ocean models are to be expected, as many aspects of these models can vary, including numerical methods, grid structure, resolution (horizontal and vertical) and parameterizations of unresolved physical processes.

In this case, investigation showed that the observed differences can be explained by higher velocities in BNAM, which resulted in particle drifts calculated by Parcels following the same general tracks as those produced with WebDrogue and Quoddy but moving further^20^. Parcels and WebDrogue both track particles based on the same kinematics formula:

$$ {\text{x}}_{{\text{t}}} = \int_{{t_{0} }}^{{t_{1} }} {v\;dt + {\text{x}}_{0} } $$

Thus, in the 2-D simulations compared^20^, it was concluded that any difference in the calculated position (x_t_) for a given time (*t*) and release position (x_0_) must be caused by differences in the velocities (*v*) imported from the ocean models (BNAM and Quoddy respectively).

BNAM and Quoddy belong to different families of ocean models. BNAM uses horizontally-structured grids, whereas Quoddy uses horizontally-unstructured grids. Wang et al.^27^ have compared surface currents derived from BNAM with mapped currents derived from surface-drifter data, finding strong correlation, which suggests that the BNAM model’s outputs are realistic for the region of interest here, at least for surface waters. We suggest that the connectivity patterns determined with Parcels herein, should be considered to better represent reality in the region around Flemish Cap in part due to this support from independent data but also due to the incorporation of vertical displacement through the use of the 3-D modeling, which is shown to potentially have a large influence on the LPT outputs for this region (Figs. 2, 3, 8).

### Physical oceanographic characteristics in the region

Lazier and Wright^28^ report the presence of a deeper extension of the Labrador Current which they call the “Deep Labrador Current” (DLC). Hall et al.^29^ show this as a possibly separate branch of the current reaching depths below 1000 m. Farther offshore, intensified toward the bottom layer, there is the Deep Western Boundary Current (DWBC), a dominant feature in the bottom layer between 2500 and 3500 m water depth. While some of the closed areas reach the influence of the DWBC, most lie at intermediate depths where the DLC predominates, and controls the connectivity among the NAFO closed areas in the Flemish Cap region. Biophysical connectivity patterns for large-sized sponges and large gorgonian corals show strong downstream interdependence with upstream closed areas on the DLC path acting as source populations for adjacent downstream closed areas. In some cases, with longer drift durations, this downstream connectivity confers redundancy on some areas. Area 2, the largest of the closed areas and recipient of water from both branches of the DLC^30,31^ (the branch through Flemish Pass and the branch rounding Flemish Cap) has the greatest redundancy and shows retention due to its larger size. We know that size is a factor here as Area 1, which is downstream of Area 1 and an order of magnitude smaller in size (Supplementary Table S5) shows no redundancy and minimal retention (Supplementary Table S1). For sea pens, in the shallower waters of Flemish Cap (still > 578 m), we were able to detect connectivity among several small closed areas (Areas 7, 8, 9, 10, 11 and 12) using both forward- and back-tracking models, that was influenced by an area where some RAFOS floats ballasted for 700 m reversed their clock-wise direction and travelled west through Flemish Pass^32^.

Our models also showed large downward vertical velocities in the deep waters below 1000 m of the continental slopes surrounding Flemish Cap and the Tail of Grand Bank (Fig. 8), which has significant implications for the benthic invertebrate fauna in this region. Current distributions at depths of 100 m and 1000 m, with horizontal velocity vectors and vertical velocity contours (Fig. 8) revealed a prevalent downward vertical velocity around Flemish Cap, with alternating areas of upward and downward velocity along the continental slope. From the horizontal velocity vectors at the two depths in the southeast of Flemish Cap (Fig. 8a,b), it is clear that the current there is dominated by the NAC in shallow water, while this situation changes in deep water and becomes DLC-dominated current. These vertical velocity maps could be used in species distribution models as environmental layers to determine their importance as habitat predictors.

Vertical velocity slices indicate that the downward velocity occurs in the upper slope areas with upward velocity in the deeper slope and offshore areas with implications for the downslope transport of organisms and nutrients from upper layers and recycling of nutrients from the deep waters. At 1000 m and deeper, vertical displacements are strong mainly due to these downward vertical velocities, and also the strong DLC along the slope. Moorings placed on Sackville Spur, Flemish Cap (Area 6) have shown that the DLC follows along isobaths between water depths of ~ 1200 and 2200 m in that area^32^. The pattern was less strong but still evident at the surface, 100 and 450 m (Fig. 8) suggesting a mechanism for vertical transport of food from the more productive surface waters to the deep sea (e.g.^14^). Such linkages have been demonstrated in numerous areas within the North Atlantic and have been suggested as being largely responsible for fuelling productive and diverse deep-sea ecosystems such as those containing corals and sponges^4^. In addition to food supply, the prominent downward vertical velocities and strong horizontal currents likely create a barrier to active larval movement to the surface from release sites on the seafloor. This adds to the several water mass layers previously described as potential barriers to larval movement from the closed areas put in place by NAFO^7^. Further, the strong baroclinic northeastward NAC mainly impacts these top layers, and the impacts of this current on the particle dispersals at the surface layer are significant, carrying the particles away from the Flemish Cap area^27^.

The DLC has been found to have significant inter-annual variability, and a weakening trend has existed for the past 20 years^33^; the weakening trend is probably linked to increasing heat content in the Labrador Sea due to weakening in winter atmospheric cooling over the past three decades^19,34^. The changes in the benthic layer over the Labrador Sea slope (at about 1000 m water depth) are found to be highly correlated with the changes in the heat content of the entire 2000 m top layer in the Labrador Sea. These and similar long-term changes in the DLC spreading along the Labrador slope are likely to have significant consequences for the connectivity of benthic bathyal fauna in this region^20^, as demonstrated here.

The interpolated EN4 data showed that all of the closed areas have maintained a stable environment over past decades, where the conditions contribute to the establishment and survival of the protected species. It may also indicate that the species are less likely to adapt to climate change due to a potential requirement for habitat stability. If bottom temperature increases in the future, which is a very high likelihood for this region, it could affect species distributions^21^. Morato et al.^21^ predicted shifts in the distribution of selected deep-sea species in the North Atlantic, including that of two large gorgonian corals that occur in the Flemish Cap region, *Acanthogorgia armata* and *Paragorgia arborea*, which were induced by future (2081–2100) climate change based on the current (1951–2000) environmental conditions. They found that both species are predicted to lose > 80% of their habitat, with *Paragorgia arborea* showing near extinction of suitable habitat (99%). The present-day temperature data collected for the basin-scale distribution of these species was 4.92 ± 2.21 °C for *A. armata* and 3.87 ± 2.26 °C for *P. arborea*, which is slightly warmer than the EN4 data from Areas 2, 4, 5 and 13 (Supplementary Fig. S6) but consistent with observational data from the cruises which are slightly elevated above EN4. Projection of bottom temperatures to above 5 °C in this region by 2081–2100 (average ± standard deviation of 4.169 ± 0.657 °C from 1951–2000 to 5.794 ± 1.152 °C by 2081–2100^21^) would require a greater acclimation for *P. arborea* than it has experienced over recent decades, with corals in Area 13 perhaps being best adapted to the projected warming due to the present and historical warmer bottom temperatures there (Supplementary Fig. S6).

In conclusion, 3-D LPT simulations for the Flemish Cap region have shown enhanced connectivity over 2-D models and unexpected, current-driven, strong (up to 1000 m +) potential for downward displacement at depth (> 450 m to 2250 m). The area closures in this region selected to protect vulnerable marine species are all at depths greater than 450 m. Despite a general lack of knowledge of larval duration and behaviour of these species^7^ preventing further, more specific incorporation of particle behaviour into our study, our results indicate that there are even greater barriers to surface movements of larvae released from the sea bed at depth than had previously been found^7^ due to downwelling at depth. High correlations between seasonal connectivity patterns suggest that the lack of information on spawning time for many of the coral and sponge species, is unlikely to change these results substantively. The current velocities create down-stream interdependence among closed areas and allow redundancy to develop in some of the areas of the network, with some of the larger areas showing retention as well as redundancy (e.g., Areas 2 and 4). At the same time, source populations for sponges in the upstream closed area (Area 6) are likely in adjacent waters of the Canadian continental shelf where additional sponge grounds are predicted to occur^10^. Potential sources for sponge larvae drifting into Area 6 (Fig. 4) have been identified as coming from the Northeast Newfoundland Slope Closure, a 55,353 km^2^ Canadian marine refuge put in place to protect corals and sponges on the continental slope from all bottom contact fishing activities (https://www.dfo-mpo.gc.ca/oceans/oeabcm-amcepz/refuges/northeastnewfoundlandslope-talusnordestdeterreneuve-eng.html). This Canadian marine refuge may support the sponge populations in the high-seas NAFO area of Flemish Cap. Collectively this information can be used to inform management decisions related to the size and placement of these closed areas, and demonstrates the added value connectivity modeling can bring in building redundancy into MPA network design, even across political boundaries. Identification of areas of upward and downward velocity (Fig. 8) have potential for use in species distribution modeling as predictors of habitat suitability.

## Methods

### Lagrangian particle simulation

The Parcels framework version 2.1 (https://www.oceanparcels.org^9,35^) was used to perform three-dimensional (3-D) passive particle tracking experiments in the northwest Atlantic. Climatological monthly–mean currents were obtained from the Bedford Institute of Oceanography North Atlantic Model (BNAM)^27,33,36,37^ ocean model over the period 1990–2015. BNAM is an eddy resolving North Atlantic Ocean model with a nominal resolution of 1/12° (approximately ~ 8 km at the Equator). It is based on NEMO 2.3 (Nucleus for European Modelling of the Ocean) and has a maximum of 50 levels in the vertical, with level thickness increasing from 1 m at the surface to 200 m at a depth of 1250 m and reaching the maximum thickness of 460 m at the bottom of the deep basins (approximately 5730 m). BNAM uses partial cell for the bottom layer, which improves the representation of the bottom layer.

We advected Lagrangian particles using the fourth-order Runge–Kutta method as the integration scheme with a time step of 20 min, and a horizontal mixing of 100 m^2^ s^−1^^9^. Optimal parameters for the number of particles, and particle spacing were determined to minimize computational time without introducing bias^20^. A single 20 min. time step was chosen for releasing and following particle trajectories to ensure that particles do not cross the grid in a single time step. Horizontal diffusion in the study region previously has been reported as 50^38^, 64^39^ and 150 m^2^ s^−1^^40^ and the spatial distribution of modeled particles did not differ markedly with applications of 100 and 200 m^2^ s^−1^^20^. Particle spacing of 0.01° was generally employed^20^ except for the calculation of back-tracked transit time distributions for the assessment of functional connectivity, where a finer spacing of 0.005° was used to allow more particles to be both released, and thus to enter the closed areas with smaller spatial size (e.g., Areas 7–9). Particles were removed from the simulation if they reached the boundary of the modeled spatial domain, delineated by the marine area bounded by the 31.4° and 73.9° W meridians and by the 40.8° and 70.5° N parallels of latitude.

### Simulation experiments

Our experiments simulated larval transport and determined how temporal and spatial ocean current changes affect dispersal. We performed three different simulation experiments aimed at determining different aspects of physical connectivity among NAFO closed areas (Fig. 1, Supplementary Table S4). Within each experiment we ran a number of scenarios evaluating different depths, northern hemisphere seasons (through extraction of associated ocean model data) and drift-time durations (Supplementary Table S4). Season and drift-time duration were based on a previous review of the life-history characteristics of the coral, sponge and sea pen species that are protected by the closures^7^ and allow model outputs to be interpreted as biophysical models for the assessment of functional connectivity. Parcels allows for larval behaviour to be incorporated into the tracking model (e.g., swimming, vertical movements), however, very little is known about the reproductive biology of the deep-sea species protected by the closed areas^7^. Consequently, we consider larvae as passive objects in order to reduce uncertainties, as such behavior is difficult to quantify and validate^41^ and the larvae of many deep-sea species have not been observed in situ.

### Vertical movements in 3-D models

The upward and downward movement of forward-tracking particles released from each of the 14 closed areas was evaluated at each of 5 depths for 3 drift durations using average and seasonal monthly-mean currents from BNAM (Supplementary Table S4). Particle spacing of 0.01° was employed for particle seeding^20^. Particles were seeded uniformly (total number of particles in brackets) at the surface (14,909), 100 m (14,909), 450 m (14,877), 1000 m (11,399) and 2250 m (1308) depths, over all of the closed areas at the initial time point, and then tracked forward under each drift duration scenario (2 weeks, 1 month and 3 months). Because the closed areas differ in surface area with the increase in depth, the number of simulated particles released in each area varied and decreased in deep water with the same uniform particle spacing. The depths were chosen such that the maximum arrival depth of the first run became the approximate particle release depth for the subsequent run. From each particle trajectory, the following were recorded: (1) maximum and minimum depths trajected to by each particle for each release depth; (2) considering the randomness of particle motion, depths reached by the first 25%, 50% and 75% of particles were recorded. In a final simulation, the impact of seasons was investigated by seeding particles at depths of surface, 100 m, 450 m, 1000 m and 2250 m across four northern hemisphere seasons, and the average depth of 50% of sorted results recorded.

### Potential source populations

For the purpose of identifying possible species source populations for those found inside the closed areas, both forward- and back-tracking simulations were utilized. Wang et al.^20^ has shown that the results from forward-tracking particles in this region are nearly identical to those produced through backtracking from the closed areas. Our experiments were run in 3-D under each of three different drift scenarios (2 weeks, 1 month and 3 months) with data extracted from an averaged (no seasonality) BNAM ocean model with particles released at 1000 m depth, which approximated the mean depth across all closed areas (Supplementary Table S5). Particle spacing of 0.01° was employed for seeding^20^. For forward-tracking, 927,621 particles at 1000 m depth were seeded uniformly over the entire model domain at the initial time, and then forward-tracked under each drift duration scenario. For back-tracking, 11,417 particles at 1000 m depth were seeded uniformly over each of the closed areas (Fig. 1c) at the initial time, and then back-tracked under each drift duration scenario. Particles whose trajectory ended in or passed over a closed area were retained, and their initial positions binned and counted on a 0.1° × 0.1° grid.

### Functional connectivity of sponges, sea pens and large Gorgonian corals

Six of the 14 closed areas (Areas 1–6) in the NAFO regulatory area on Flemish Cap and the Tail of Grand Bank (Fig. 1) were put in place to protect sponge ground ecosystems from significant adverse impacts of bottom contact fishing. These assemblages live at depths from 700 to 1400 m on the deep slopes of Flemish Cap and Grand Bank^42^. All known sponge larvae are lecithotrophic, and they are unable to capture food from their surroundings, therefore they are not expected to swim to surface waters to feed. Sponge larvae are capable of actively swimming but most of the time they are thought to rest in a vertical position and drift passively with the water current^24^. A large majority of larvae were found to stay around the parental habitat in the demersal water layer^43,44^, and in our models, we released particles from the seabed of each of the six closed areas.

Eight of the 14 closed areas (Areas 2, 7–12 and 14) were closed to protect sea pens in the relatively shallower waters of Flemish Cap, with the species forming a distinct community on sandy and silt bottoms in the study area^42^. There is some information on the spawning season for some species or their congeneric representatives^7^ but in general little is known about the reproductive biology of the 13 sea pen VME indicator taxa known to occur on Flemish Cap^45^. Areas 2, 4, 5 and 13 provide conservation and protection for large gorgonian corals with at least 14 shallow- and deep-water species occurring in the study area^45^. Nothing is known of the pelagic larval duration (PLD) or the vertical position in the water column for these sea pen larvae or for the larvae of the large gorgonian corals. Three different durations (2 weeks, 1 month and 3 months) were considered to encompass all likely scenarios^7^.

Functional 3-D connectivity among closed areas containing specific deep-sea species was assessed with a PLD of 2 weeks, 1 month and 3 months using averaged and seasonal monthly-mean currents from BNAM (Supplementary Table S4). Particles were released at depths that reflected the on-bottom depths for each closed area for those areas designed to protect the same species (Supplementary Tables S5, S6). The minimum, middle and maximum depths of the mean depth ranges for the combined areas determined particle release depths (Supplementary Tables S5, S6). For all scenarios, the total number of released particles were initiated inside one of the closed areas (uniformly positioned) and the models run under each scenario in both forward and backward modes. The forward models used the 0.01° particle spacing. By uniformly seeding all of the closed areas at a finer grid size (0.005°, see Lagrangian Particle Simulation above) and running back-tracking models, potential sources in the network of other closed areas could be identified for all closed areas except for Area 1, which had no downstream closed area to receive from. Supplementary Table S6 shows the particle numbers released for the models run for sponges, sea pens and large gorgonian corals at different depths.

To quantify connectivity based on forward- and backward-tracking particle trajectories the percentage of particles passing over and/or terminating within a closed area, including retentions (particles terminating in the area they were released from), were calculated^46^. For backtracked models only, we calculated Pearson’s r correlation coefficient to statistically compare the numbers of particles passing over or terminating in each closed area for seasonal and averaged oceanographic models. This was only done for the sponges and the sea pens, where biological information on spawning season was inferred, and hence called for seasonal models to be developed. No information on spawning time of the large gorgonian corals in the region was available to justify application of seasonal models for that functional group.

For both forward- and back-tracking models we also calculated the transit time distribution (TTD)^47^ which shows the number of days it takes for each particle to arrive in each closed area (excluding retentions), creating a histogram of arrival times. The data were then normalized by the total number of particles arriving in each area^48^. To be able to directly compare TTD for both forward- and back-tracking models, we applied the same particle spacing of 0.005 degrees and a 20 min. time step. Considering the limited pelagic larval duration for the coral and sponge species in this area^7^, in general, the faster that particles reach an area we assume a higher probability of successful settlement due to higher survivorship of larvae, provided that there is sufficient time for metamorphosis to occur. The percentage of particles passing over a closed area reflects the quantity of particles that reach the area, and the larger this proportion, we assume a greater the likelihood for the species to successfully recruit to the next generation.

### Water mass characteristics of the closed areas

The water mass characteristics for each of the closed areas can be used to identify water masses influencing the benthic species and provide insight into their physiological niche. Temperature and salinity data from 1975 to 2017 from EN4 (https://www.metoffice.gov.uk/hadobs/en4/^49^) were used to examine bottom water mass characteristics for the investigated 14 NAFO closed areas (Fig. 1). In addition, the temperature and salinity values extracted from the back-tracked connectivity pathways from the BNAM model were examined for stability over the drift duration. Original data sources of EN4 include the World Ocean Database (WOD^50^), the Global Temperature-Salinity Profile Programme (from 1990), and ARGO profiling data. The data were objectively analyzed onto a 1° grid with 42 vertical levels for each month. Those data were linearly interpolated onto a 0.03° grid for the Flemish Cap region with temperature and salinity values extracted for each of the 14 areas (Fig. 1). Observational temperature and salinity data (CTD and/or SBE39) from scientific cruise surveys conducted by the Bedford Institute of Oceanography (BIO) from the closed areas were used to validate the interpolated EN4 data in closed Areas, 2, 3, 4, 5, 6, 10 and 12.

## Supplementary information

Supplementary Information.

## Data Availability

Lagrangian particle simulator (Parcels) is freely available at https://www.oceanparcels.org; EN4 dataset for temperature and salinity from 1975 to 2017 can be obtained from the website https://www.metoffice.gov.uk/hadobs/en4/; climatological monthly–mean currents from BNAM and vertical velocity layers (Fig. 8a,b) are available on Mendeley Data^51^.
