# Supplementary material for: 3-D ocean particle tracking modeling reveals extensive vertical movement and downstream interdependence of closed areas in the northwest Atlantic
Source: Sci Rep. 2020 Dec 8;10:21421. doi: 10.1038/s41598-020-76617-x (PMC7722887; doi:10.1038/s41598-020-76617-x)
Supplement: Supplementary file 1 — Supplementary Information. [file 41598_2020_76617_MOESM1_ESM.pdf]

Supplementary Information Figures and Tables

3-D Ocean Particle Tracking Modeling Reveals Extensive Vertical Movement and Downstream Interdependence of Closed Areas in the Northwest Atlantic

S. Wang[1], E. L. Kenchington[1*], Z. Wang[1], I. Yashayaev[1], A. J. Davies[2]

[1]Department of Fisheries and Oceans Canada, Bedford Institute of Oceanography, 1 Challenger Drive, Dartmouth, NS, B2Y 4A2, Canada

[2]University of Rhode Island, Department of Biological Sciences, Center for Biotechnology and Life Sciences, 120 Flagg Road, Kingston, RI 02881, USA

*Correspondence to Ellen.Kenchington@dfo-mpo.gc.ca

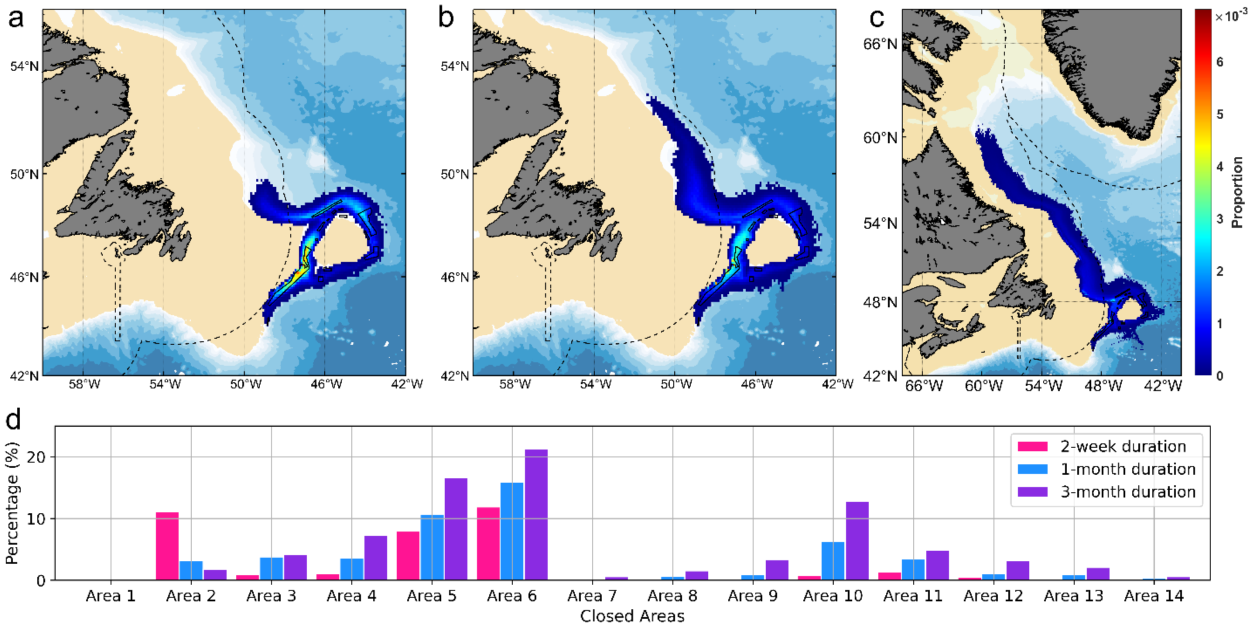

Figure S1. Potential Population Sources. Spatial distribution of the number of released particles ending in and passing over each 0.1° × 0.1° grid cell with particles released at 1,000 m depth within each of the closed areas (Fig. 1c) that reached that depth (Supplementary Table S5) and back-tracked for three drift durations: a) 2 weeks, b) 1 month and c) 3 months. d) the proportion of released particles (1,000 m) entering each closed area with three drift duration scenarios. Note that for Area 1 there were no closed areas downstream and so no possibility of particles arriving there except through retention. Dashed lines on the maps indicate boundaries of national jurisdictions downloaded from the Maritime Boundaries Geodatabase, version 11, available online at https://www.marineregions.org/, https://doi.org/10.14284/382. The maps were generated using bathymetry and coastline data produced and made publically available by the NOAA National Centers for Environmental Information (NCEI). The ETOPO1 Ice Surface (doi:10.7289/V5C8276M) arc-minute global relief model of the Earth's surface (https://www.ngdc.noaa.gov/mgg/global/) was used to generate bathymetry and the Global Self-consistent, Hierarchical, High-resolution Geography Database (GSHHG; https://www.ngdc.noaa.gov/mgg/shorelines/gshhs.html) was used to produce co-ordinates for a high-resolution coastline and both plotted using Matlab version 9.5 software (http://www.mathworks.com) with the M_Map mapping package (version 1.4 m, created by R. Pawlowicz, https://www.eoas.ubc.ca/~rich/map.html).

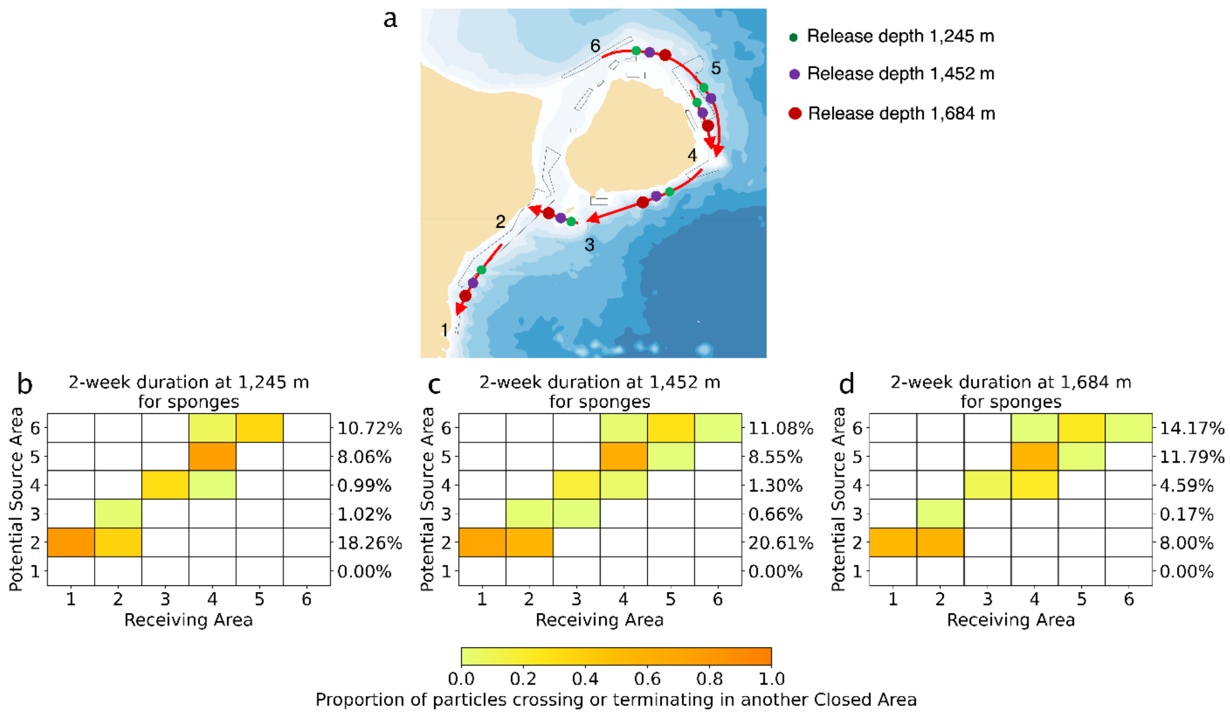

Figure S2. Functional Connectivity Among Areas Closed to Protect Sponges. a) Back-tracked connectivity pathways for particles released at 3 depths in each of the 6 areas closed to protect large-size sponges (Areas 1- 6) showing chain-linking and minimal redundancy in Area 4. Drift durations were 2 weeks. The closed circles over the lines indicate particles can reach another area when released from this depth. The map was generated using bathymetry and coastline data produced and made publically available by the NOAA National Centers for Environmental Information (NCEI). The ETOPO1 Ice Surface (doi:10.7289/V5C8276M) arc-minute global relief model of the Earth's surface (https://www.ngdc.noaa.gov/mgg/global/) was used to generate bathymetry and the Global Self-consistent, Hierarchical, High-resolution Geography Database (GSHHG; https://www.ngdc.noaa.gov/mgg/shorelines/gshhs.html) was used to produce co-ordinates for a high-resolution coastline and both plotted using Matlab version 9.5 software (http://www.mathworks.com) with the M_Map mapping package (version 1.4 m, created by R. Pawlowicz, https://www.eoas.ubc.ca/~rich/map.html); b-d) The proportion of modeled particles released from each of the 6 areas closed to protect large-sized sponges (Receiving Areas; Areas 1, 2, 3, 4, 5, 6) and passing over or terminating in another area closed to protect large-size sponges (Potential Source Areas) for 1,245 m, 1,452 m and 1,684 m release depths. For each Potential Source Area the percentage of the total number of particles released (from all releases from all Receiving Areas) are provided. Those values include particles that crossed, terminated or were retained in the Potential Source Area. Drift durations were 2 weeks using average currents from the ocean model. Note that in the backward tracking models Area 1 is unable to receive particles as there are no closed areas downstream.

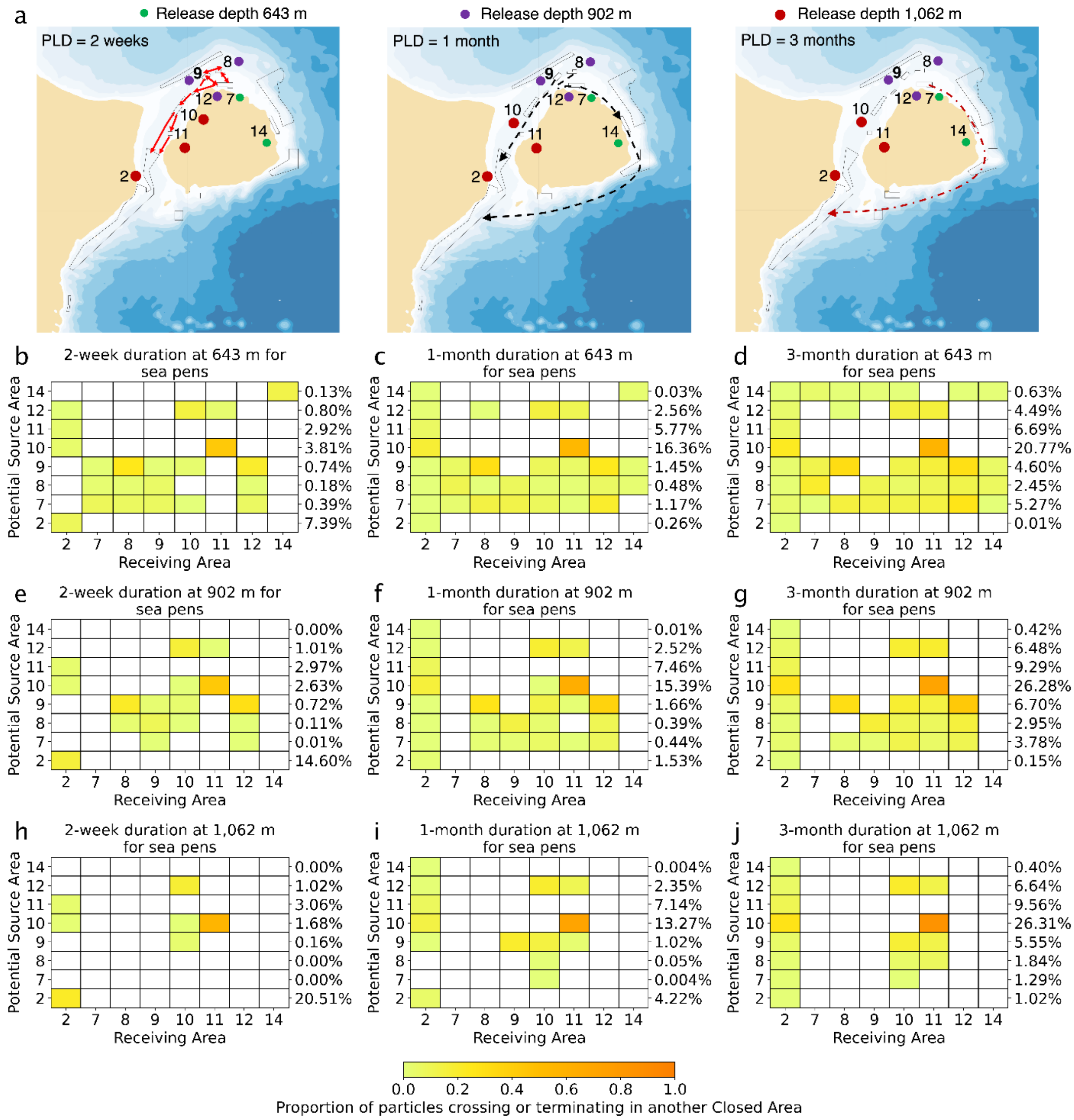

Figure S3. Functional Connectivity Among Areas Closed to Protect Sea Pens. a) Back-tracked connectivity pathways for particles released at 3 depths in each of the 8 areas closed to protect sea pens (Areas 2, 7, 8, 9, 10, 11, 12, 14). Drift durations were 2 weeks, 1 month, and 3 months. The closed circles near the number code of some of the closed areas denotes that particles from this area are only released at this depth due to the depth of the closure, and the line indicates where particles can reach another closed area when released from this depth. b-j) The proportion of modeled particles released from each of the 8 areas closed to protect sea pens (Receiving Areas; Areas 2, 7, 8, 9, 10, 11, 12, 14) and passing over or terminating in another area closed to protect sea pens (Potential Source Areas). For each Potential Source Area the percentage of the total number of particles released (from all Receiving Areas) are provided. Those values include particles that crossed, terminated or were retained in the Potential Source Area. Particle release depths were 643 m (b-d), 902 m (e-g) and 1,062 m (h-j) with drift durations of 2 weeks (b, e, h), 1 month (c, f, i), and 3 months (d, g, j). The maps were generated using bathymetry and coastline data produced and

made publically available by the NOAA National Centers for Environmental Information (NCEI). The ETOPO1 Ice Surface (doi:10.7289/V5C8276M) arc-minute global relief model of the Earth's surface (https://www.ngdc.noaa.gov/mgg/global/) was used to generate bathymetry and the Global Self-consistent, Hierarchical, High-resolution Geography Database (GSHHG; https://www.ngdc.noaa.gov/mgg/shorelines/gshhs.html) was used to produce co-ordinates for a high-resolution coastline and both plotted using Matlab version 9.5 software (http://www.mathworks.com) with the M_Map mapping package (version 1.4 m, created by R. Pawlowicz, https://www.eoas.ubc.ca/~rich/map.html).

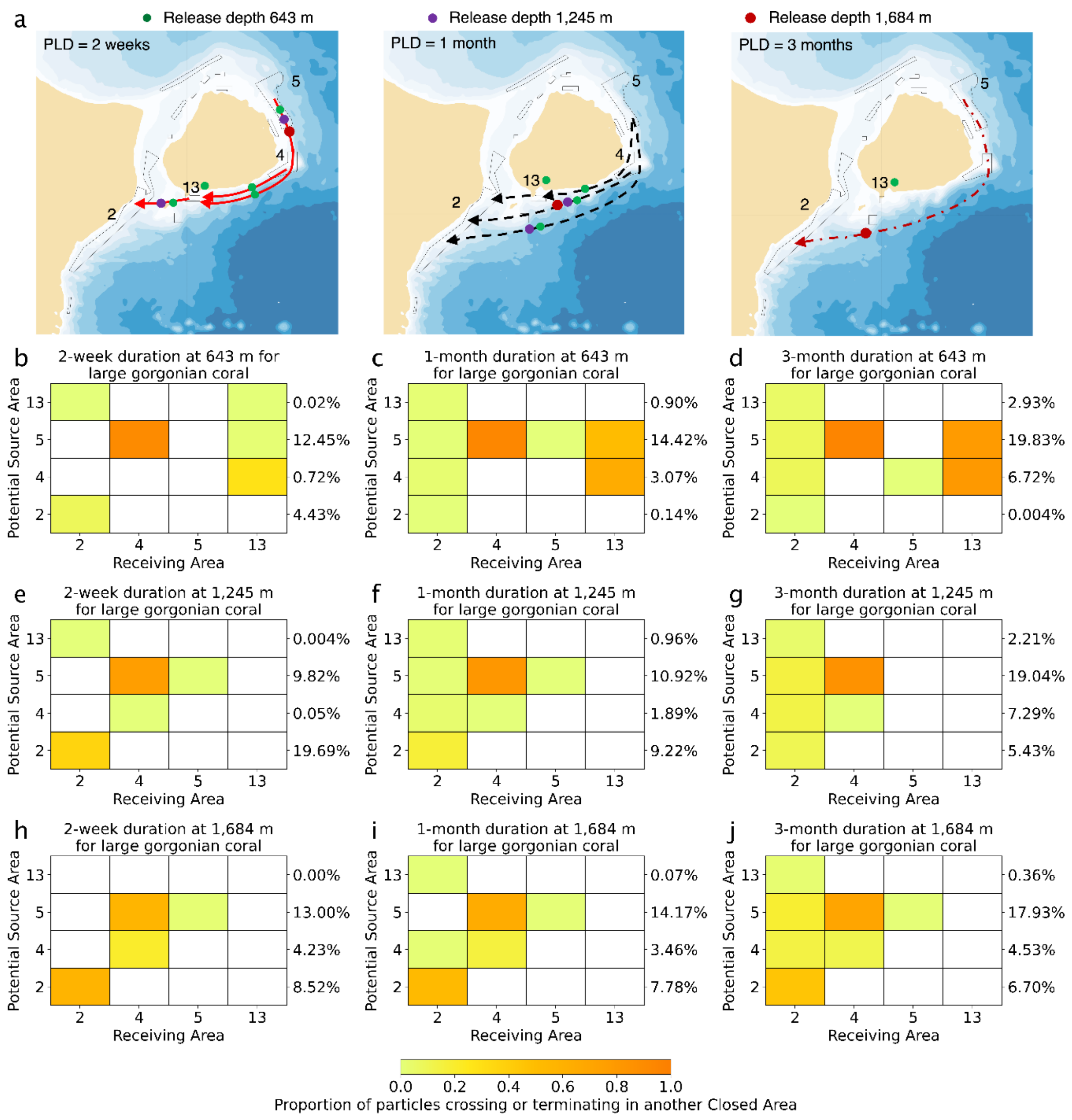

Figure S4. Functional Connectivity Among Areas Closed to Protect Large Gorgonian Corals. a) Back-tracked connectivity pathways for particles released at 3 depths in each of the 4 areas closed to protect large gorgonian corals (Areas 2, 4, 5, 13). Drift durations were 2 weeks, 1 month, and 3 months. The closed circles near the number code of some of the closed areas denotes that particles from this area are only released at this depth due to the depth of the closure, and the line indicates where particles can reach another closed area when released from this depth. (b-j) The proportion of modeled particles released from each of the 4 areas closed to protect large gorgonian corals (Receiving Areas; Areas 2, 4, 5, 13) and passing over or terminating in another area closed to protect large gorgonian corals (Potential Source Areas). For each Potential Source Area the percentage of the total number of particles released (from all Receiving Areas) are provided. Those values include particles that crossed, terminated or were retained in the Potential Source Area. Particle release depths were 643 m (b-d), 1,245m (e-g) and 1,684 m (h-j) with drift durations of 2 weeks (b, e, h), 1 month (c, f, i), and 3 months (d, g, j). The maps were generated using bathymetry and coastline data produced and made publically available by the NOAA National Centers for Environmental

Information (NCEI). The ETOPO1 Ice Surface (doi:10.7289/V5C8276M) arc-minute global relief model of the Earth's surface (https://www.ngdc.noaa.gov/mgg/global/) was used to generate bathymetry and the Global Self-consistent, Hierarchical, High-resolution Geography Database (GSHHG; https://www.ngdc.noaa.gov/mgg/shorelines/gshhs.html) was used to produce co-ordinates for a high-resolution coastline and both plotted using Matlab version 9.5 software (http://www.mathworks.com) with the M_Map mapping package (version 1.4 m, created by R. Pawlowicz, https://www.eoas.ubc.ca/~rich/map.html).

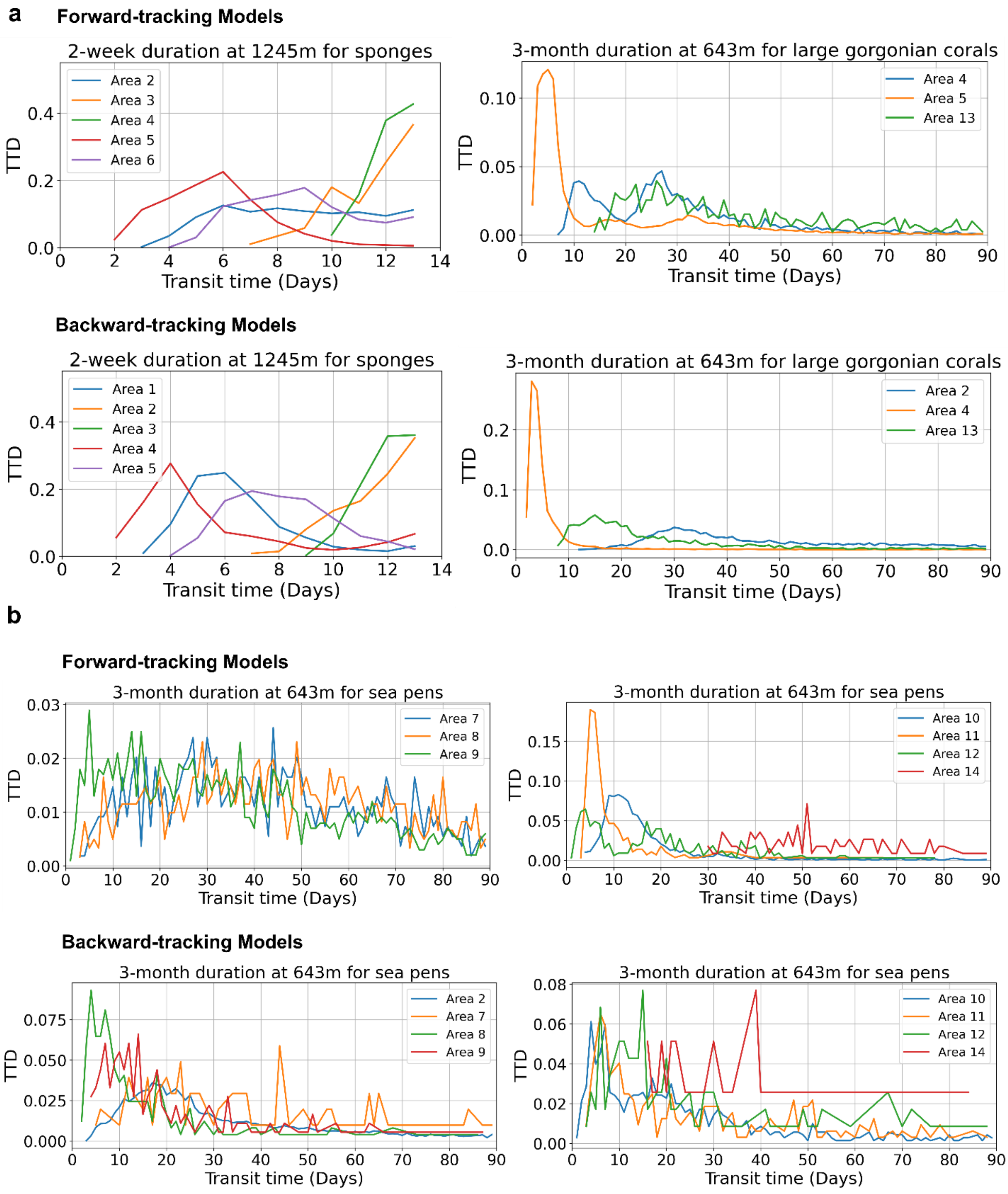

Figure S5. Transit time distributions (TTD). Particles released from the closed areas and tracked in forward and backward models to another closed area. a) TTDs for areas closed to protect sponges (left panels) and large gorgonian corals (right panels); b) TTDs for areas closed to protect sea pens. Closed areas with no or few particles terminating or passing over are not shown. TTDs for particles released from the minimum depth of the mean depth ranges for the combined areas for each functional group are shown. Insets: 'Areas' indicate Release Areas.

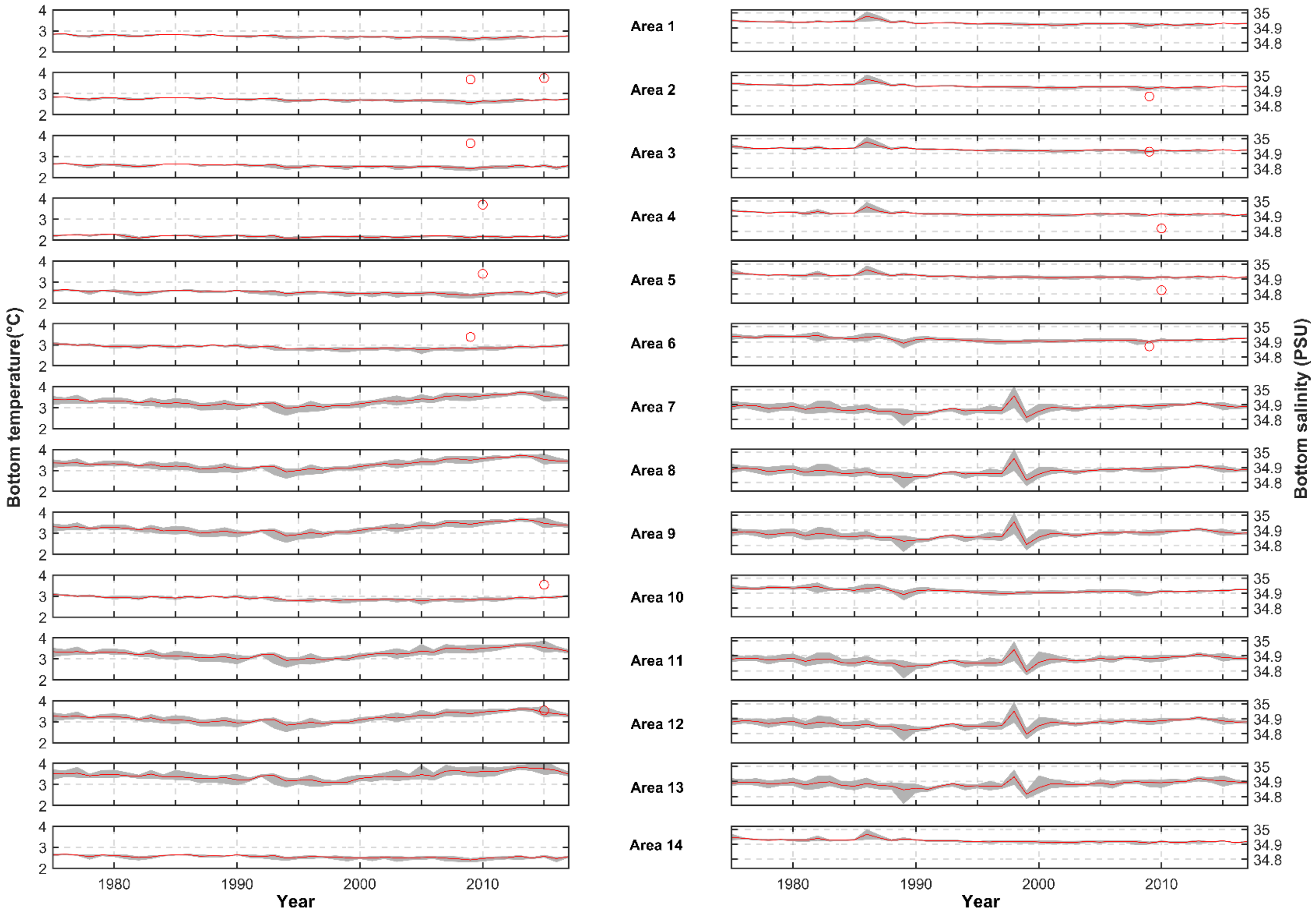

Figure S6. Historical Bottom Conditions of Temperature and Salinity in each Closed Area. Variation of bottom temperature (left panel) and salinity (right panel) in each closed area obtained from the interpolated EN4 data. The shaded areas denote the range of those data in each area within each year, and red lines are the averaged data. Red circles denote averaged observational data by year from independent scientific missions conducted by the Bedford Institute of Oceanography and were not included in the EN4 model.

Table S1. Functional Connectivity Among Areas Closed to Protect Sponges. Drift trajectories for forward-modeled particle releases from within each closed area (Fig. 1c) showing closed areas where particles connected (passed over and/or ended) as well as those with endpoints within the release area (particle retention), by release depth and seasons (Summer, Autumn, Average specified if not found under all scenarios) for areas closed to protect deep-sea sponges. Drift duration was 2 weeks. *Indicates connection also found in back-tracking models using averaged oceanographic data; **Indicates connection only found in back-tracking models using averaged oceanographic data.

**Release depth**	**Particles passing over/ending in closed areas from release area**	**Endpoints within the release area (retention)**
1,245 m	Area 2 to Area *1 (Summer, Autumn, Average)	Areas *2, *4 (Average)
	Area 3 to Area *2 (Summer, Autumn, Average)	Area 2 (Summer)
	Area 4 to Area *3 (Summer, Autumn, Average)	Area 2 (Autumn)
	Area 5 to Areas *4 (Summer, Autumn, Average), 3 (Summer)	
	Area 6 to Areas *5, *4 (Summer, Autumn, Average)	
1,452 m	Area 2 to Area *1 (Summer, Autumn, Average)	Area 1 (Average)
	Area 3 to Area *2 (Summer, Autumn, Average)	Areas *2, *4, *5 (Average)
	Area 4 to Area *3 (Summer, Autumn, Average)	Areas **3, **6 (Average)
	Area 5 to Area *4 (Summer, Autumn, Average)	Areas 2, 4, 5 (Autumn)
	Area 6 to Areas *5, *4 (Summer, Autumn, Average)	Areas 2, 5 (Summer)
1,684 m	Area 2 to Area *1 (Summer, Autumn, Average)	Areas 2, 3, 4, 5 (Autumn)
	Area 3 to Area *2 (Summer, Autumn, Average)	Areas 2, 4, 5 (Summer)
	Area 4 to Area *3 (Summer, Autumn, Average)	Area 3 (Average)
	Area 5 to Area *4 (Summer, Autumn, Average)	Areas *2, *4, *5 (Average)
	Area 6 to Area *5 (Summer, Autumn, Average), **4 (Average)	Area **6 (Average)

Table S2. Functional Connectivity Among Areas Closed to Protect Sea Pens. Drift trajectories for forward-modeled particle releases from within each closed area (Fig. 1c) showing closed areas where particles passed over/ended as well as those with endpoints within the release area (Particle Retention), by release depth, drift duration (2 weeks, 1 month, and 3 months) and seasons (Spring, Summer, Winter, Average specified) for areas closed to protect sea pens. *Indicates connection also found in back-tracking models using averaged oceanographic data; **Indicates connection only found in back-tracking models using averaged oceanographic data.

**Drift duration**	**Particles passing over/ending in closed areas from release area**	**Endpoints within the release area (retention)**
Release depth 643 m		
2 weeks	Area 7 to Areas *8, *9, *12 (Spring, Summer, Winter, Average), 10 (Summer) **10 (Average)	Areas *2, *7, *8, *9, *14 (Spring, Winter, Average)
	Area 8 to Areas *7, *9, *12 (Spring, Summer, Winter, Average) 14 (Spring) 10 (Summer)	Areas 2, 7, 8, 11, 14 (Summer)
	Area 9 to Areas *8, *10, *12 (Spring, Summer, Winter, Average) 7 (Summer) **7 (Average)	
	Area 10 to Areas *2, *11 (Spring, Summer, Winter, Average)	
	Area 11 to Area *2 (Spring, Summer, Winter, Average)	
	Area 12 to Areas *10, *11 (Spring, Summer, Winter, Average) 2 (Summer) 9 (Winter) **2 (Average)	
1 month	Area 7 to Areas *8, *9, *10, *12 (Spring, Summer, Winter, Average), *2 (Summer, Winter, Average), 11 (Summer), 14 (Winter), **11 (Average)	Areas 2, 9, 14 (Spring)
	Area 8 to Areas *7, *9, *10, *12, *14 (Spring, Summer, Winter, Average), 2, 11 (Summer), **2, **11 (Average)	Areas *2, *7, *8, *14 (Summer, Average)
	Area 9 to Areas *2, *7, *8, *10, *11, *12, *14 (Spring, Summer, Winter, Average)	Areas 2, 14 (Winter)

	Area 10 to Areas *2,*11 (Spring, Summer, Winter, Average)	
	Area 11 to Area *2 (Spring, Summer, Winter, Average)	
	Area 12 to Area *2, *10, *11 (Spring, Summer, Winter, Average), 9 (Winter),**8 (Average)	
	Area 14 to Area **2 (Average)	
3 months	Area 7 to Areas *2, *8, *9, *10, *11, *12 (Spring, Summer, Winter, Average), *14 (Winter, Average)	Area *2 (Spring, Summer, Winter, Average)
	Area 8 to Areas *2, *7, *9, *10, *11, *12, *14 (Spring, Summer, Winter, Average)	Area 7 (Winter)
	Area 9 to Areas *2, *7, *8, *10, *11, *12, *14 (Spring, Summer, Winter, Average)	Area **7, **14 (Average)
	Area 10 to Areas *11, *2 (Spring, Summer, Winter, Average)	
	Area 11 to Area *2 (Spring, Summer, Winter, Average)	
	Area 12 to Areas *2, *10, *11 (Spring, Summer, Winter, Average), 9 (Winter),**8 (Average)	
	Area 14 to Area *2 (Spring, Summer, Winter, Average), 7, 10, 11, 12 (Summer), **7, **8, **9, **10, **12 (Average)	
Release depth 902 m		
2 weeks	Area 7 to Areas **9, **12 (Average)	Areas *2, *8, *9 (Spring, Summer, Winter, Average)
	Area 8 to Areas *9 (Spring, Summer, Winter, Average), *12 (Summer, Average), **10 (Average)	Area 10 (Spring, Winter)<br>Area **10 (Average)
	Area 9 to Areas *8 (Summer, Winter, Average) 10, 12 (Spring, Summer) **10, **12 (Average)	
	Area 10 to Areas *2, *11 (Spring, Summer, Winter, Average)	

	Area 11 to Area *2 (Spring, Summer, Winter, Average)	
	Area 12 to Areas *10 (Spring, Summer, Winter, Average), 9 (Spring), 11(Summer), **11 (Average)	
1 month	Area 7 to Areas **2, **8, **9, **10, **11, **12 (Average)	Area *2 (Spring, Summer, Winter, Average)
	Area 8 to Areas *9, *10, *12 (Spring, Summer, Average), 2, 7 (Summer), 9, 14 (Winter), **2 (Average)	Area 9 (Spring)
	Area 9 to Areas *8, *10, *12 (Spring, Summer, Winter, Average), 2, 11 (Spring, Summer, Winter), 14 (Spring, Average) **2, **11 (Average)	Area *8, *10 (Average)
	Area 10 to Areas *2, *11 (Spring, Summer, Winter, Average)	
	Area 11 to Area *2 (Spring, Summer, Winter, Average)	
	Area 12 to Areas *2, *10, *11 (Spring, Summer, Winter, Average), 9 (Spring)	
	Area 14 to Area **2 (Average)	
3 months	Area 7 to Areas **2, **8, **9, **10, **11, **12 (Average)	Area *2 (Spring, Summer, Winter, Average)
	Area 8 to Areas *2, *9, *10, *12 (Spring, Summer, Winter, Average), 7 (Spring, Summer, Winter), *11 (Spring, Summer, Average) 14 (Summer, Winter)	
	Area 9 to Areas *2, *8, *10, *11, *12 (Spring, Summer, Winter, Average), 14 (Spring, Average), 7 (Spring, Summer)	
	Area 10 to Areas *2, *11 (Spring, Summer, Winter, Average)	
	Area 11 to Area *2 (Spring, Summer, Winter, Average)	

	Area 12 to Areas *2, *10, *11 (Spring, Summer, Winter, Average), 9 (Spring)	
	Area 14 to Area **2 (Average)	
Release depth 1,062 m		
2 weeks	Area 9 to Area 8 (Winter), **10 (Average)	Area *2 (Spring, Summer, Winter, Average)
	Area 10 to Areas *2, *11 (Spring, Summer, Winter, Average)	Area 10 (Summer, Winter)<br>Area **10 (Average)
	Area 11 to Area *2 (Spring, Summer, Winter, Average)	
	Area 12 to Area **10 (Average)	
1 month	Area 7 to Area **10 (Average)	Area *2 (Spring, Summer, Winter, Average)
	Area 8 to Area **10 (Average)	Area **9 (Average)
	Area 9 to Areas 8 (Winter),**2,**10,**11 (Average)	
	Area 10 to Area *2, *11 (Spring, Summer, Winter, Average)	
	Area 11 to Area *2 (Spring, Summer, Winter, Average)	
	Area 12 to Areas **2, **10, **11 (Average)	
	Area 14 to Area **2 (Average)	
3 months	Area 7 to Areas **2, **10 (Average)	Area *2 (Spring, Summer, Winter, Average)
	Area 8 to Areas **2, **10, **11 (Average)	
	Area 9 to Areas 2 (Summer), 8 (Winter), **2, **10, **11 (Average)	
	Area 10 to Area *2, *11 (Spring, Summer, Winter, Average)	
	Area 11 to Area *2 (Spring, Summer, Winter, Average)	
	Area 12 to Areas **2,**10,**11 (Average)	

	Area 14 to Area **2 (Average)	

Table S3. Functional Connectivity Among Areas Closed to Protect Large Gorgonian Corals. Drift trajectories for forward-modeled particle releases from within each closed area showing closed areas where particles passed over/ended as well as those with endpoints within the release area (particle retention), by release depth and drift duration (2 weeks, 1 month, and 3 months) for areas closed to protect large gorgonian corals. Average values from the ocean model were used. *Indicates connection also found in back-tracking models; **Indicates connection only found in back-tracking models.

**Release Depth**	**Drift duration**	**Particles passing over/ending in closed areas from release area**	**Endpoints within the release area (retention**
643 m	2 weeks	Area 4 to Area *13	Areas *2, *13
		Area 5 to Areas *4, *13	
		Area 13 to Area **2	
	1 month	Area 4 to Areas *2, *13	Areas *2,**5
		Area 5 to Areas *2, *4, *13	
		Area 13 to Area *2	
	3 months	Area 4 to Area *2, **5, *13	Area *2
		Area 5 to Area *2, *4, *13	
		Area 13 to Area *2	
1,245 m	2 weeks	Area 4 to Area 13	Areas *2, *4,**5
		Area 5 to Areas *4, 13	
		Area 13 to Area **2	
	1 month	Area 4 to Area *2, 13	Areas *2, **4,**5
		Area 5 to Areas *2, *4, 13	
		Area 13 to Area **2	
	3 months	Area 4 to Area *2, 13	Areas *2, **4
		Area 5 to Areas *2, *4, 13	
		Area 13 to Area **2	
1,684 m	2 weeks	Area 5 to Area *4	Areas *2, *4,**5
	1 month	Area 4 to Area *2	Areas *2, *4,**5
		Area 5 to Area 2, *4, 13	
		Area 13 to Area **2	
	3 months	Area 4 to Area *2	Areas *2, *4,**5
		Area 5 to Area *2, *4, 13	
		Area 13 to Area **2	

Table S4. Overview of 3-D Particle Tracking Experiments Performed.

Experimental objective	Particle release depth	Season for BNAM extracts	Drift durations
Vertical movement	Surface, 100 m, 450 m, 1,000 m, 2,250 m	Average, Spring, Summer, Autumn, Winter	2 weeks, 1 month and 3 months
Potential source population	1,000 m	Average	2 weeks, 1 month and 3 months
Functional connectivity	Benthic habitats (see Supplementary Table S6)	Forward tracking models: Average, Spring, Summer, Autumn, Winter according to conservation target spawning season if known<br><br>Backward tracking models: Average	2 weeks, 1 month and 3 months

Table S5. Spatial Characteristics of NAFO Closed Areas to Protect VMEs on Flemish Cap and Grand Bank, Northwest Atlantic.

Closed Area	Minimum depth (m)	Maximum depth (m)	Mean depth (m)	Surface area (km^2)
1	1,174	1,917	1,516	143.8
2	483	2,211	1,262	5,421.4
3	921	2,598	1431	307.6
4	567	2,754	1,272	1,357.6
5	938	2,688	1,776	2,878.6
6	1,224	1,952	1,562	987.5
7	590	718	650	258.0
8	905	1,088	978	97.9
9	876	1,120	992	127.7
10	1,013	1,177	1,127	315.6
11	910	1,132	1,067	60.5
12	922	1,003	958	35.1
13	523	924	666	338.4
14	578	688	627	239.1

Table S6. Model Scenarios for Assessing Functional Connectivity Among Areas Closed to Protect Sponges, Sea Pens and Large Gorgonian Corals. The minimum, middle and maximum depths of the mean depth ranges for the combined areas determined particle release depths.

Model	Closed Areas	Drift Duration	Particle Release Depth (m)	Particle Numbers	
				Forward-Tracking Models	Backward-Tracking Models
Sponges	Areas 1-6	2 weeks	1,245	8,472	33,915
			1,452	5,373	21,519
			1,684	2,998	11,995
Sea pens	Areas 2, 7-12, 14	2 weeks, 1 month, 3 months	643	7,203	28,868
			902	6,374	25,521
			1,062	5,667	5,667
Large gorgonian corals	Areas 2, 4, 5, 13	2 weeks, 1 month, 3 months	643	11,686	46,794
			1,245	7,091	28,356
			1,684	2,768	11,073
